# Mortality and adverse events of hemoadsorption with CytoSorb® in critically ill patients: A systematic review and meta‐analysis of randomized controlled trials

**DOI:** 10.1111/aas.14115

**Published:** 2022-07-18

**Authors:** Marc Heymann, Raoul Schorer, Alessandro Putzu

**Affiliations:** ^1^ Division of Anesthesiology, Department of Acute Medicine Geneva University Hospitals Geneva Switzerland

**Keywords:** adverse events, CytoSorb, Hemoadsorption, hemoperfusion, mortality, safety

## Abstract

**Background:**

The effects and safety of extracorporeal hemoadsorption with CytoSorb® in critically ill patients with inflammatory conditions are controversial.

**Methods:**

We performed a systematic review with meta‐analysis and trial sequential analysis (TSA) of randomized‐controlled trials to assess the mortality and safety of CytoSorb® therapy in critically ill patients with inflammatory conditions. Electronic databases were searched up to April 2022. The primary outcome was mortality at longest follow‐up and secondary outcomes included various adverse event (AE) outcomes. Conflict of interest and funding of each trial were assessed. We calculated relative risk (RR) and 95% confidence interval (CI).

**Results:**

Fourteen published (*n* = 764) and 4 unpublished (*n* = 111) trials were included. Eight trials were performed in medical ICU patients and 10 in complex cardiac surgery. Ten trials had significant industrial funding or an author conflict of interest. Hemoadsorption with CytoSorb® was associated with higher mortality at latest follow‐up (16 trials, *n* = 807, 120 of 402 [29.85%] patients in the CytoSorb® group vs. 98 of 405 [24.20%] patients in the control group, RR = 1.24 [95% CI, 1.04–1.49], *p* = .02, [TSA‐adjusted CI, 0.92–1.68]) and at 30‐days or in‐hospital (11 trials, *n* = 727; RR = 1.41 [95% CI, 1.06–1.88], *p* = .02, [TSA‐adjusted CI, 0.44–4.62]). Only one trial reported the definition of adverse event, while detailed results were reported in 3 trials; the risk of adverse events was not higher with CytoSorb®. Certainty of evidence ranged from low to very low.

**Conclusion:**

Low certainty of evidence showed that the use of CytoSorb® might increase mortality in critically ill patients with inflammatory conditions. Adverse events were frequent but underreported and not systematically evaluated. Industrial funding and conflict of interest were common. Considerable uncertainty about the findings does not allow firm conclusions and suggests a need for high‐quality randomized trials to clarify mortality and adverse events related to CytoSorb®.

**Editorial Comment:**

Hemoadsorption with CytoSorb® have been used in critically ill patients despite lack of high quality data from RCTs suggesting any patient‐important benefits. The findings from this systematic review and meta‐analysis suggests an increased risk of adverse events including mortality. With no apparent benefits and at the same time risk of harm, use of hemoadsorption with CytoSorb® in daily clinical practice cannot be recommended at this time.

## 1 INTRODUCTION

The use of extracorporeal hemoadsorption has been suggested as a potential treatment in states of severe inflammatory response such as sepsis, acute respiratory distress syndrome (ARDS), cardiac surgery and more recently in coronavirus disease (COVID‐19).^1^ The pathophysiology of those diseases involves complex cellular and biochemical interactions primarily mediated by cytokines. Removing those proteins from the blood has therefore been hypothesized to be an effective way to improve clinical outcome. Despite the existence of several blood purification devices and their experimental use in the last 30 years in sepsis and septic shock, their effectiveness is inconclusive and these therapies have not entered routine clinical practice.^2^, ^3^ Polymyxin B immobilized fiber column hemoadsorption was evaluated in a cumulative population of more than 1100 septic patients without quality evidence supporting a beneficial effect on survival.^2^, ^3^, ^4^ Furthermore, much of the research in extracorporeal blood purification therapies in sepsis has been industry‐driven.^5^

CytoSorb® (CytoSorbents, Monmouth Junction, NJ, USA) is a medical device consisting of a biocompatible and hemocompatible porous polymer sorbent bead technology.^6^ It can be integrated into an extracorporeal pump circuit including renal replacement therapy, extracorporeal membrane oxygenation (ECMO), and heart‐lung machines.^6^ CytoSorb® reduces the concentration of pro‐ and anti‐inflammatory cytokines and pathogen‐associated molecular pattern molecules in vitro.^7^, ^8^ Theoretically, those effects should translate in vivo and thus mitigate physiological shock and improve clinical outcome. Various case series and observational studies reported beneficial effects such as shock reversal and improved mortality in various patient populations, without significant safety concerns.^9^, ^10^, ^11^, ^12^, ^13^ Results from propensity score matching studies are conflicting: some studies found beneficial effects on mortality,^14^ while others found no significant effects on hemodynamic stabilization and mortality.^15^, ^16^ The use of this device was also suggested in high risk cardiac surgery and a potential outcome benefit was reported in non‐randomized trials.^17^, ^18^

However, results of randomized controlled trials (RCTs) using CytoSorb® have so far been disappointing. The largest RCT performed to date found a higher mortality in septic patients on hemoadsorption.^19^ Furthermore, recent small randomized trials in patients on extracorporeal membrane oxygenation (ECMO) found a higher mortality rate with CytoSorb®.^20^, ^21^ These results raised some questions regarding mortality and safety related to the use of this device.^22^, ^23^ We therefore performed a systematic review with meta‐analysis of randomized trials evaluating the performance and safety of CytoSorb® therapy. We hypothesized that the use of CytoSorb® hemoadsorption would increase mortality and adverse events in adult critically ill patients.

## 2 MATERIALS AND METHODS

The present systematic review was conducted in compliance with the PRISMA (Preferred Reporting Items Systematic Reviews and Meta‐Analysis) guidelines (PRISMA 2020 checklist, Table S1) and Cochrane methodology and according to a pre‐published protocol (PROSPERO database, CRD42021259447).^24^, ^25^ This study had no funding and authors did not have any conflicts of interest.

### 2.1 Search strategy

Two investigators (AP and MH) independently searched MEDLINE, EMBASE, and the Cochrane Central Register of Clinical Trials for appropriate articles up to April 27, 2022 for relevant articles. Search strategies are reported in the Supplementary Methods S1. For unpublished trials, we searched Clinicaltrials.gov and the World Health Organization International Clinical Trials Registry Platform. Bibliographies of retrieved trials and of relevant systematic and narrative reviews were also screened. No language restriction was enforced.

### 2.2 Study selection

References obtained from searches were first independently examined at the abstract level by two authors (AP and MH) and then collected as full‐text articles if potentially relevant. Eligible studies met the following PICOS criteria: (1) Population: adult critically ill patients, including patients undergoing major surgery; (2) Intervention: extracorporeal hemoadsorption with CytoSorb®; (3) Comparison intervention: standard treatment only or sham hemoadsorption; (4) Outcome: any primary or secondary outcome of the present review (see below); (5) Study design: randomized controlled trial. Trials with populations overlapping that of a previously included article and pediatric studies were excluded. Two authors (AP and MH) independently assessed selected studies for the final analysis, with disagreements resolved by consensus

### 2.3 Data abstraction

One author (AP) extracted data from eligible studies and stored them into a predefined database. Another author (MH) verified the data, with divergences resolved by consensus. Sources of significant clinical heterogeneity were extracted (e.g., study design, clinical setting, inclusion and exclusion criteria, blood purification regimen). Complete‐case analysis was used to assess outcomes data. If the article did not include data on mortality, the corresponding author was contacted for further data by one author (AP). In case of no reply to the first e‐mail, a second one was sent 5 to 15 days later.

### 2.4 Outcomes

The primary outcome was mortality at longest follow‐up available. Secondary outcomes were 30‐days or in‐hospital mortality and adverse events. The adverse events outcomes were: (a) number of patients with at least one serious adverse event (SAE); (b) number of patients with at least one adverse event (AE) of any grade; (c) number of patients with an AE leading to death; (d) total number of SAEs; (e) total number of AEs; (f) total number of non‐serious AEs, and (g) total number of device‐related AEs. Adverse events outcomes were defined by study authors and extracted as reported in each study.

### 2.5 Conflict  of interest

Possible financial conflict of interest, suggested by significant commercial funding of the study or author financial (direct) conflict of interest, were assessed by two authors (MH and AP). We categorized each study as: “notable concern about conflict of interest,” “no notable concern about conflict of interest,” or “unclear concern about conflict of interests.” In case of unclear conflict of interest, we tried to resolve the item through the assessment of other papers published by the study authors. We reported details on author conflict of interest (e.g., lead or corresponding authors, other authors) and the stage of the trial to which they contributed (design, conduct, analysis, reporting). We reported details on funding and sponsorship of the trial and whether the role of the funding body was reported for study design, conduct, analysis, and reporting.^25^ Finally, we assessed non‐financial (indirect) conflict of interest for each author post‐hoc.^25^, ^26^ We assessed various items including multiple publications on extracorporeal therapies, acknowledged extracorporeal therapy expert, holding a position in or consulting for a relevant committee/board/group related to extracorporeal therapies, and obvious personal belief in hemoadsorption therapy.^26^

### 2.6 Risk of bias

The risk of bias of each RCT included was evaluated according to the Cochrane Risk of Bias 2 tool by two authors (MH and AP).^25^ The assessment was performed at the outcome level (mortality and AE outcomes). The following items were evaluated for each trial: bias arising from the randomization process, bias due to deviations from intended interventions, bias due to missing outcome data, bias in measurement of the outcome, and bias in selection of the reported result. The overall risk‐of‐bias judgment was categorized as: low risk of bias, some concerns, and high risk of bias.

### 2.7 Statistical analysis

Individual trial and summary results of dichotomous data were reported as relative risk (RR) with 95% confidence interval (CI). Rates of adverse events that can occur multiple time in the same patient were compared using rate ratio with 95% CI.^25^ A *p*‐value smaller or equal to .05 was considered statistically significant. Given the anticipated substantial clinical heterogeneity, we used an inverse variance random‐effects model in all analyses. To assess between‐study heterogeneity, we used Cochran's Q statistic and the *I* ^2^ statistic. An *I* ^2^ equal to 50% and *p*‐value equal to .10 were threshold values indicating significant heterogeneity. We assessed publication and reporting bias using funnel plots when 10 or more trials per comparison were included.^25^ Meta‐analyses and forest plots were computed using Review Manager software (RevMan, version 5.4, The Cochrane Collaboration, 2020).

We conducted a trial sequential analysis (TSA) for dichotomous outcomes with the purpose of maintaining an overall 5% risk of type I error and a 20% risk of type II error (power of 80%). A post hoc relative risk increase (RRI) of 20% was assumed. We derived the control event proportion from the actual dataset. The resulting required information size (RIS) was further diversity (*D* ^2^)‐adjusted. In case of *D* ^2^ = 0, we performed a sensitivity analysis using a *D* ^2^ = 25%. The analysis was performed using the TSA Viewer software (Version 0.9.5.10 Beta. Copenhagen Trial Unit, Centre for Clinical Intervention Research, Rigshospitalet, Copenhagen, Denmark).

To test the robustness of the effect estimates and to explain heterogeneity for primary outcomes, we used sensitivity analyses and subgroup analyses in all cases where at least 2 RCTs reported the outcome. The following sensitivity analyses for primary outcomes were planned: using a fixed‐effects model; using risk difference in case of several zero events; including only published trials. Some subgroup analyses were planned based upon the following hypotheses: (1) trials with high risk of bias or some concerns will show a different treatment effect than trials with low risk of bias; (2) trials with notable conflict of interest will show greater treatment effect than trials without financial conflict of interest; (3) participants with sepsis, ARDS, or undergoing complex surgery will have a different treatment effect than patients without those conditions; (4) published trials will show treatment effects distinct from unpublished trials. The *p*‐value for the comparison between groups was calculated; a *p*‐value smaller or equal to .05 was considered statistically significant.

Protocol amendments from the original protocol are reported in the Supplementary Methods S2.

### 2.8 Quality of evidence

The certainty of the body of evidence for each outcome was assessed using the grading of recommendations assessment, development and evaluation (GRADE) framework.^27^, ^28^ The certainty of evidence was categorized as very low, low, moderate, or high based on study limitations (risk of bias), imprecision, inconsistency, indirectness, publication bias, and large magnitude of effect.

### 2.9 Unpublished trials

To decrease the risk of publication bias, we decided post‐hoc to include data of unpublished studies on CytoSorb® use. Corresponding authors of protocols of eligible trials listed on clinical trials registers up to August 5, 2021 were contacted for further data. We contacted authors of RCTs that were reported as ongoing, completed, unknown, or stopped. The corresponding authors were contacted by one author (AP) by e‐mail and if no answer was received a second e‐mail was sent. We asked for unpublished material or information on the trial, together with data on the primary outcome.

## 3 RESULTS

### 3.1 Systematic search

**FIGURE 1:**
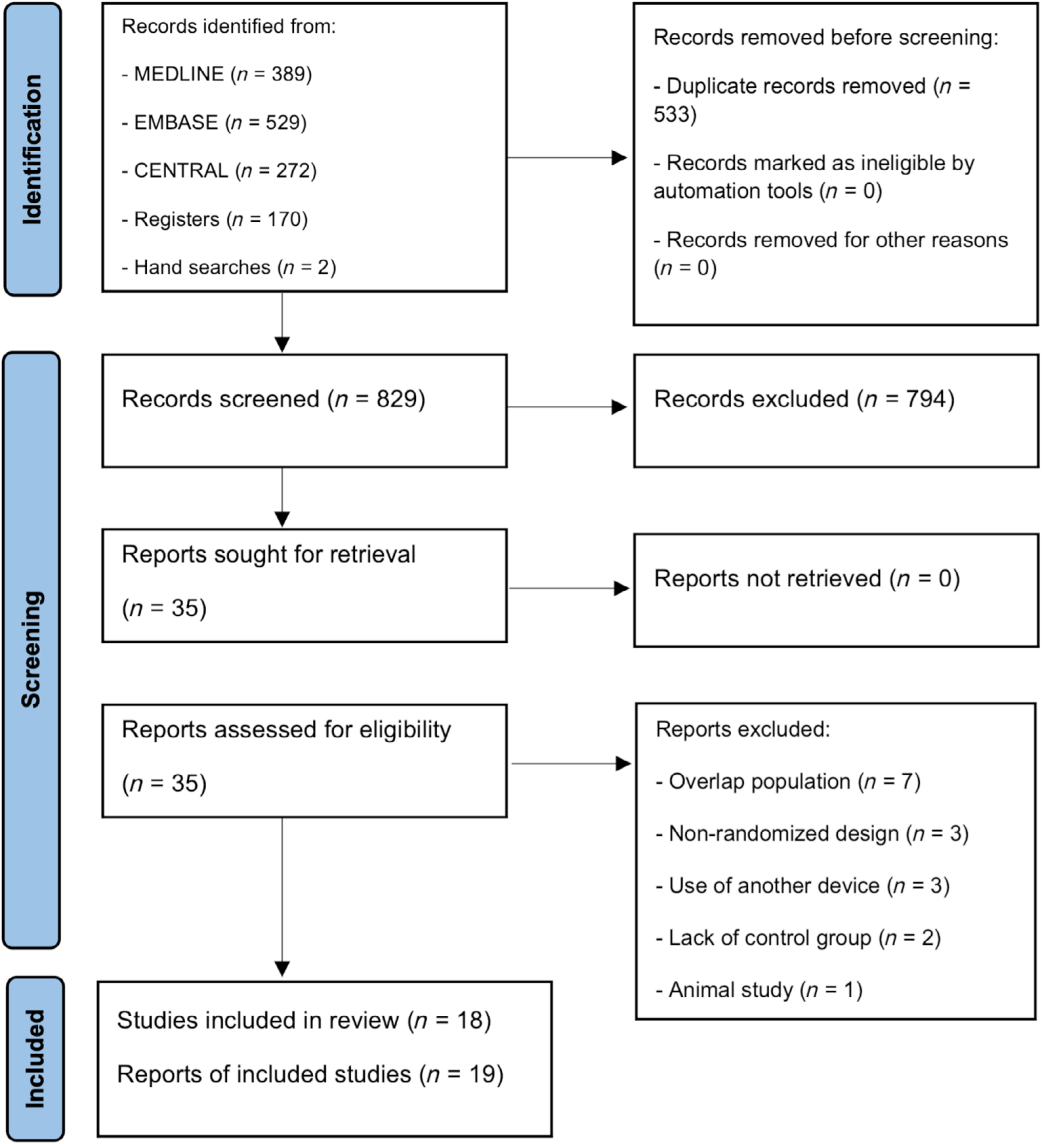
Flow diagram for the selection of studies

The systematic search in online databases produced 1362 potential titles and abstracts from database and hand searches (Figure 1). Thirty reports were identified for review, and after exclusion of ongoing trials and inadequate reports (Table S2), we included 19 reports from 18 unique trials with a total of 866 patients.^19^, ^20^, ^21^, ^29^, ^30^, ^31^, ^32^, ^33^, ^34^, ^35^, ^36^, ^37^, ^38^, ^39^, ^40^, ^41^, ^42^, ^43^

### 3.2 Study characteristics

The characteristics of the included studies are shown in Table 1 and Table S3.

**TABLE 1:** Studies characteristics

Trial	Country	Design	*N*	Population	CytoSorb® regimen	Control	Latest follow‐up
Published trials							
Asch 2021^37^	Germany	Single‐center	20	Infective endocarditis undergoing cardiac surgery	CytoSorb® incorporated in the CPB circuit. Postoperatively, integrated in a hemodialysis circuit. Treatment duration: CPB time + 24 h (cartridge change every 8 h, 4 in total).	Conventional therapy (CPB without CytoSorb®)	In‐hospital
Bernardi 2016^35^	Austria	Single‐center	37	Elective CABG, valve surgery, or combined procedure with an expected CPB duration of more than 2 h	CytoSorb® incorporated in the CPB circuit	Conventional therapy (CPB without CytoSorb®)	30 days
Diab 2022^39^	Germany	Multi‐center	202	Cardiac surgery for infective endocarditis	CytoSorb® incorporated in the CPB circuit	Conventional therapy (CPB without CytoSorb®)	30 days
Garau 2019^31^	Germany	Single‐center	40	Elective CABG, AVR, or a combined procedure with an expected CPB time of more than 2 h	CytoSorb® incorporated in the CPB circuit	Conventional therapy (CPB without CytoSorb®)	—
Gleason 2019^32^	USA	Multi‐center	46	Elective complex cardiac surgery with expected CPB duration equal or longer than 3 h	CytoSorb® incorporated in the CPB circuit	Conventional therapy (CPB without CytoSorb®)	30 days
Hawchar 2019^29^	Hungary	Single‐center	20	Intubated patients with suspected septic shock of medical origin	CytoSorb® incorporated in a blood pump circuit using a renal replacement device. Anticoagulation: heparin. Hemodialysis catheter inserted into a central vein. Treatment duration: 24 h.	Conventional therapy	Unclear
Holmén 2022^45^	Sweden	Single‐center	19	Cardiac surgery for infective endocarditis	CytoSorb® incorporated in the CPB circuit	Conventional therapy (CPB without CytoSorb®)	—
Poli 2019^30^	Switzerland	Single‐center	30	Elective cardiac surgery with expected long CPB duration and deemed at high risk of postoperative complications	CytoSorb® incorporated in the CPB circuit	Conventional therapy (CPB without CytoSorb®)	In‐hospital
Schädler 2017^19^	Germany	Multi‐center	97	Severe sepsis or septic shock a in the setting of acute lung injury or ARDS	CytoSorb® either used alone in hemoperfusion mode or incorporated in the CCVH/CVVHD circuit if renal replacement therapy was indicated. Anticoagulation: systemic heparin or regional citrate. Treatment duration: 6 h per day, up to 7 consecutive days	Conventional therapy	60‐days
Stockmann 2022^38^	Germany	Single‐center	49	COVID‐19 associated vasoplegic shock requiring norepinephrine, elevated C‐reactive protein, and indication for kidney replacement therapy	CytoSorb incorporated in the CVVHD circuit and replaced every 24 h. Treatment duration: 3–7 days according to the discretion of the treating physicians.	Conventional therapy (CVVHD without CytoSorb®)	ICU mortality
Supady 2021^20^	Germany	Single‐center	34	Severe ARDS related to SARS‐CoV‐2 infection receiving venovenous ECMO	CytoSorb® incorporated into the ECMO circuit. Treatment duration: 72 h (cartridge change every 24 h).	Conventional therapy (ECMO without CytoSorb®)	90 days
Supady 2022^21^	Germany	Single‐center	41	Extracorporeal cardiopulmonary resuscitation	CytoSorb incorporated into the ECMO circuit and replaced every 24 h. Treatment duration: 72 h.	Conventional therapy (ECMO without CytoSorb®)	30 days
Taleska Stupica 2020^34^	Slovenia	Single‐center	40	Elective complex cardiac surgery with an expected CPB time of more than 1.5 h	CytoSorb® incorporated in the CPB circuit	Conventional therapy (CPB without CytoSorb®)	1 year
Wagner 2019^33^	Czech Republic	Single‐center	28	Complex cardiac surgery (Ross operation 93%, David operation 7%)	CytoSorb® incorporated in the CPB circuit	Conventional therapy (CPB without CytoSorb®)	3 months
Unpublished trials							
NCT03145441 (ongoing)^41^	Hungary	Single‐center	51	Heart transplantation without any medical or mechanical circulatory support, sepsis, acute kidney or liver injury, or prolonged hospitalization before transplantation	CytoSorb® incorporated in the CPB circuit	Conventional therapy (CPB without CytoSorb®)	30 days
NCT03523039 (ongoing)^40^	Switzerland	Single‐center	21	Post‐cardiac arrest syndrome (need for a vasoconstrictor, elevated serum lactate) and time to return of spontaneous circulation higher than 25 minutes	CytoSorb® integrated in a hemoperfusion circuit. Anticoagulation: regional heparin‐protamine. Treatment duration: 12 to 24 h	Conventional therapy	ICU mortality
NCT04361526 (stopped)^42^	Spain	Single‐center	2	Acute onset of moderate to severe COVID‐19 ARDS needing ventilation support	CytoSorb® integrated in a hemoperfusion circuit. Anticoagulation: systemic heparin. Hemodialysis catheter inserted in a central vein. Treatment duration: 72 h (cartridge change every 24 h)	Conventional therapy	NR
NCT04518969 (ongoing)^43^	Belgium	Single‐center	9	COVID‐19 ARDS needing intubation	CytoSorb® integrated in a CVVHD circuit. Treatment duration: 4 days (cartridge change: two changes after 12 h, every 24 h after)	Conventional therapy	NR

Abbreviations: ARDS, acute respiratory distress syndrome; CABG, coronary artery bypass graft; CPB, cardiopulmonary bypass; CVVHD, continuous veno‐venous hemodialysis; ECMO, extracorporeal membrane oxygenation.

The mean age of study participants ranged between 52 and 72 years, while the proportion of female ranged between 7% and 35%. Ten trials were performed in on‐pump complex cardiac surgery (range of mean EuroSCORE II between 3 and 20), where the CytoSorb® device was integrated in the cardiopulmonary bypass machine.

Eight trials were performed in medical ICU patients with various hyperinflammatory conditions: 4 in COVID‐19 ARDS, 2 in sepsis and septic shock, 1 in post‐cardiac arrest syndrome, and 1 in extracorporeal cardiopulmonary resuscitation.

We requested further information on mortality from 22 corresponding authors. We received interim data from 3 unpublished trials (*n* = 81) and 1 early stopped trial (*n* = 2). A review author (AP) had a video conference with a CytoSorbents delegate in order to obtain more data about ongoing trials: no unpublished data was received.

### 3.3 Risk of bias

**FIGURE 2:**
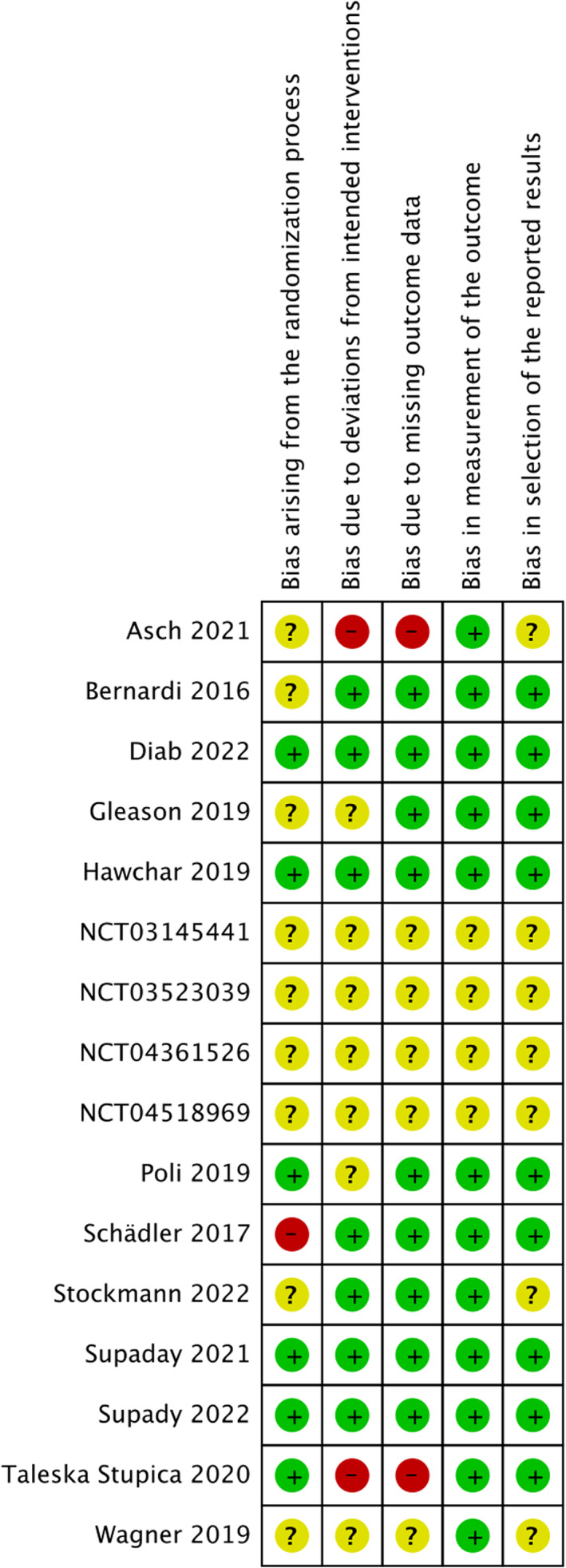
Risk of bias summary for mortality: review authors judgments about each risk of bias item for each included study

Regarding mortality, 4 trials were considered to carry a low risk of bias in all bias domains,^20^, ^21^, ^29^, ^39^ 9 trials were judged to raise some concerns in at least one domain, and 3 trials were judged at high risk of bias in at least one domain (Figure 2).^19^, ^34^, ^37^

Regarding AE outcomes, 6 trials raised some concerns in at least one domain^29^, ^32^, ^35^, ^38^, ^44^, ^45^ and 4 trials were judged at high risk of bias in at least one domain (Figure S1).^19^, ^31^, ^37^, ^39^ Three trials did not follow an intention‐to‐treat design.^31^, ^34^, ^37^ One trial excluded from the analysis patients who had severe hypotension or repeated clotting of the hemodialysis circuit integrated with CytoSorb® (4 of 15 patients).^37^

### 3.4 Conflict  of interest

All but a single published trial^37^ reported details on author financial conflict of interest and funding sources (Table S4). Twelve trials were judged to have notable concern about conflict of interest, 2 trials had no concerns,^33^, ^34^ and 4 had unclear status.^37^, ^41^, ^42^, ^43^

Eight trials were financially supported by the CytoSorb® manufacturer.^19^, ^21^, ^30^, ^31^, ^32^, ^35^, ^39^, ^45^ A trial record reported an employee of CytoSorbents corporation as “study director”;^46^ this was not reported in the manuscript of the published study.^32^

Nine trials reported the presence of financial conflict of interest in at least one study author.^19^, ^20^, ^21^, ^29^, ^30^, ^32^, ^35^, ^38^, ^39^ Twenty‐three authors reported some kind of conflict of interest with the CytoSorb® manufacturer: 4 first authors, 6 last authors, and 13 co‐authors.

Ten trials had at least 1 author judged to have nonfinancial conflict of interest. Eleven unique authors were judged to have nonfinancial conflict of interest: 11 had multiple publications and 9 were deemed acknowledged expert in the field.

### 3.5 Mortality

**FIGURE 3:**
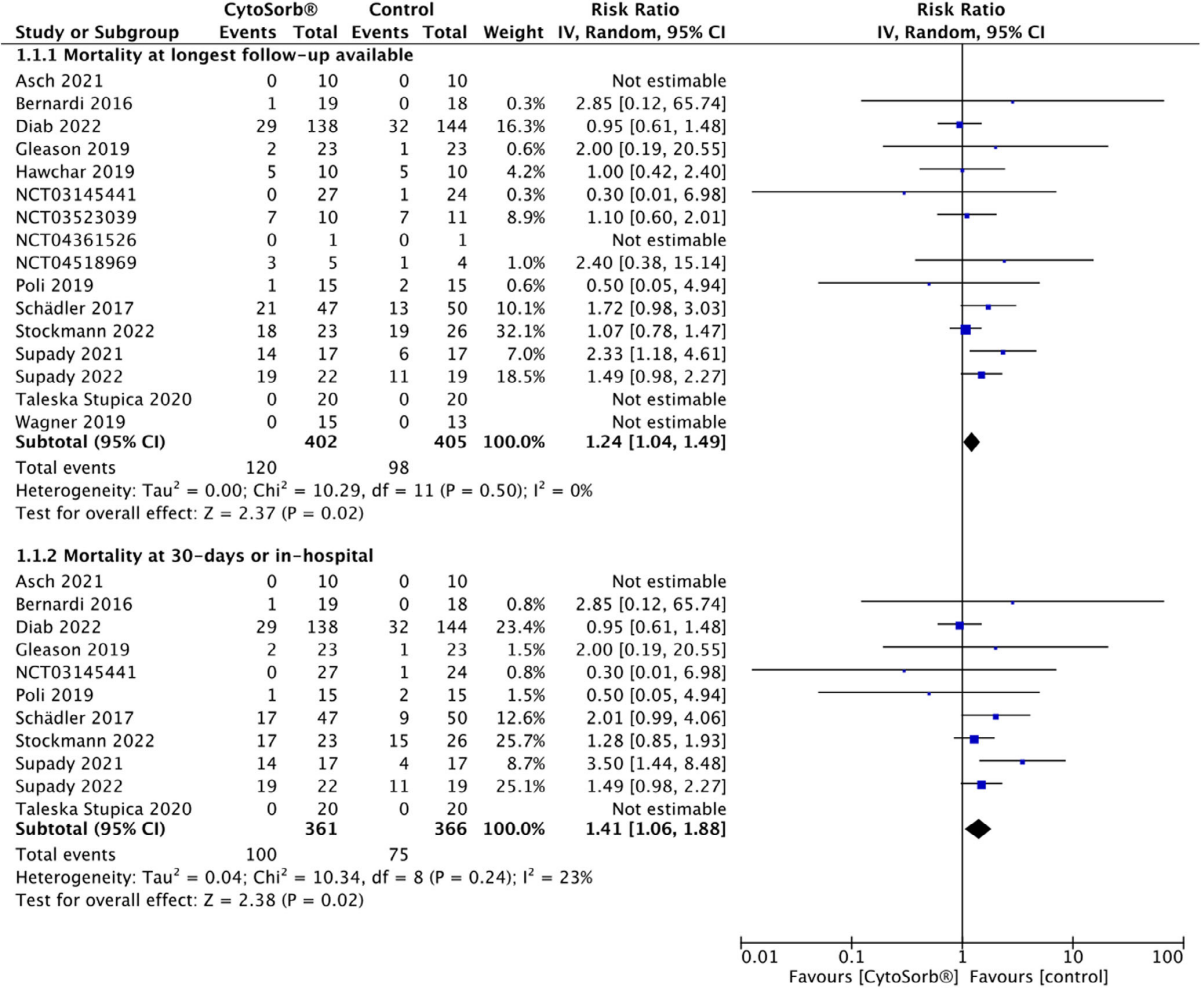
Forest plot of the relative risk of mortality at longest follow up available and at 30‐days or in‐hospital with CytoSorb® hemoadsorption and control therapy

The use of hemoadsorption with CytoSorb® was associated with a higher mortality at longest follow‐up available compared to the control group (16 trials, *n* = 807, 120 of 402 [29.85%] patients in the CytoSorb® group vs. 98 of 405 [24.20%] patients in the control group, RR = 1.24 [95% CI, 1.04 to 1.49], *p* = .02, *I* ^2^ = 0%, follow‐up: ICU to 1‐year, low certainty). The CytoSorb® device was further associated with an increase in mortality at 30‐days (11 trials, *n* = 727; RR = 1.41 [95% CI, 1.06–1.88], *p* = .02, *I* ^2^ = 23%, low certainty) (Figure 3).

**FIGURE 4:**
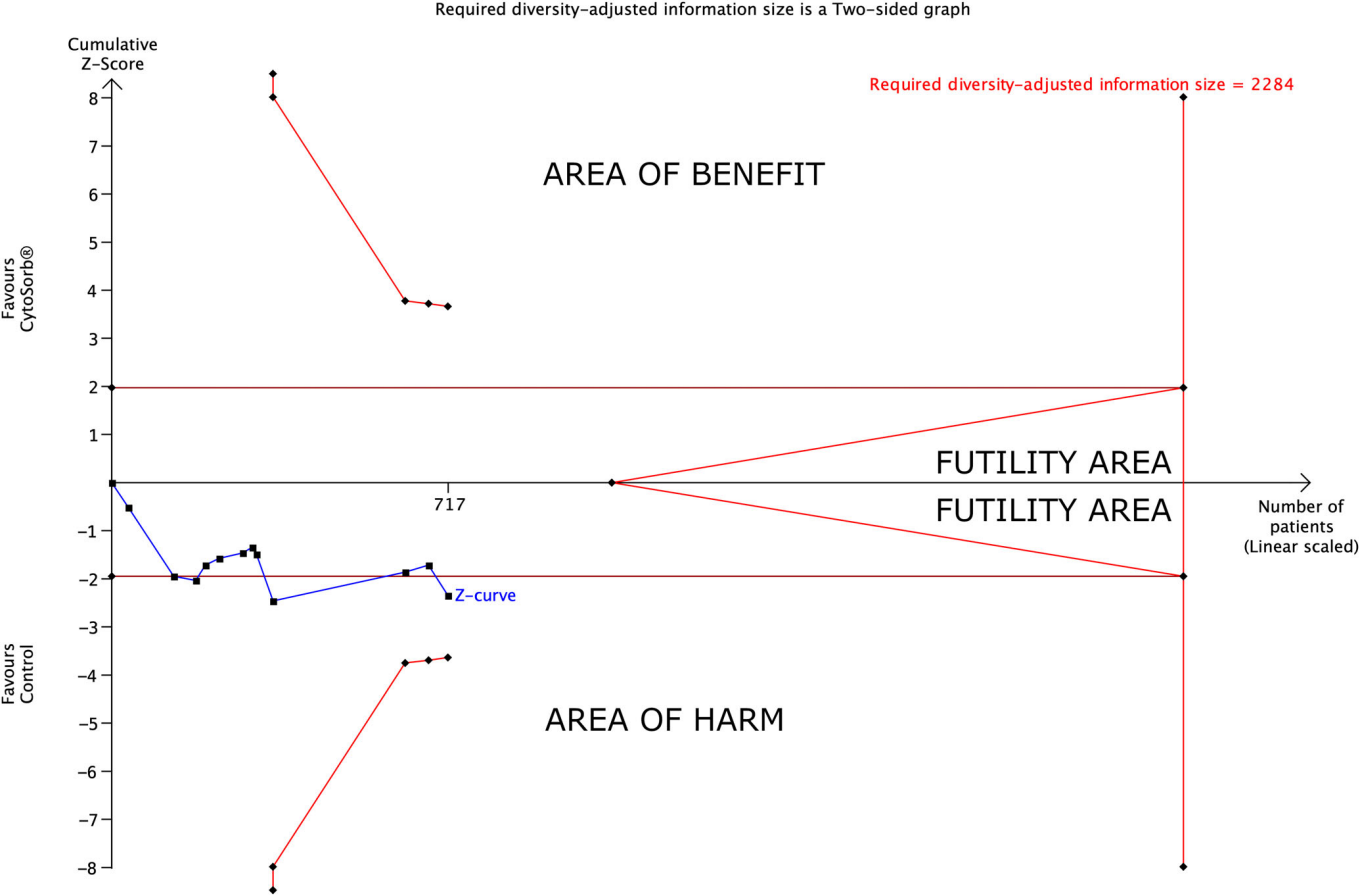
Trial sequential analysis for mortality at longest follow‐up available with CytoSorb® hemoadsorption and control therapy (TSA‐adjusted CI = 0.92 to 1.68, type I error = 5%, type II error = 20%, relative risk increase = 20%, diversity = 0%)

Trial sequential analysis found that 35% of the required information size had been accrued and TSA‐adjusted CI was 0.92–1.68 (*D* ^2^ = 0%, RIS = 2284) (Figure 4). At 30 days, the TSA‐adjusted CI was 0.44–4.62 (*D* ^2^ = 40%, RIS = 4697, RIS accrued = 15%).

No significant statistical interaction was found (*p* = .15) between mortality in medical ICU versus complex cardiac surgery patients (8 medical ICU trials, 273 patients, 149 deaths, RR = 1.34 [95% CI, 1.08–1.67], *p* = .009; 8 complex cardiac surgery trials, 534 patients, 69 deaths, RR = 0.95 [95% CI, 0.62–1.44], *p* = .80) (Figure S2). Exploratory subgroup analyses according to different medical conditions are reported in the supplement (Figure S3).

The direction of effect for subgroup and sensitivity analyses generally concurred with those of the primary analysis (Table S5). The funnel plot did not suggest pubblication bias (Figure S4).

The certainty of evidence for mortality at longest follow‐up available was judged to be low (Table 2) due to downgrading for risk of bias and imprecision. Details of the GRADE assessment are in the supplement (Table S6).

**TABLE 2:** Summary of findings table

Outcomes	Relative effect (95% CI)	№ of participants (studies)	Certainty of the evidence (GRADE)
Mortality at longest follow‐up available	Relative risk 1.24 (1.04–1.49)	807 (16 RCTs)	⨁⨁◯◯ Low
Mortality at 30‐days	Relative risk 1.41 (1.06–1.88)	727 (11 RCTs)	⨁⨁◯◯ Low
Patients with at least one serious adverse event	Relative risk 1.42 (0.87–2.33)	210 (4 RCTs)	⨁◯◯◯ Very low
Patients with at least one adverse event	Relative Rrsk 1.09 (0.98–1.21)	299 (6 RCTs)	⨁◯◯◯ Very low
Adverse event leading to death	Relative risk 0.99 (0.19–5.06)	76 (2 RCTs)	⨁◯◯◯ Very low
Total number of serious adverse events	Rate ratio 1.18 (0.86–1.63)	173 (3 RCTs)	⨁◯◯◯ Very low
Total number of adverse events	Rate ratio 0.99 (0.86–1.15)	524 (6 RCTs)	⨁◯◯◯ Very low
Total number of non‐serious adverse events	Rate ratio 0.79 (0.62–1.01)	173 (3 RCTs)	⨁◯◯◯ Very low
Total number of device‐related adverse events	Rate ratio 2.90 (0.70–12.05)	246 (6 RCTs)	⨁◯◯◯ Very low

*Notes*: *GRADE Working Group grades of evidence*High certainty: we are very confident that the true effect lies close to that of the estimate of the effect.Moderate certainty: we are moderately confident in the effect estimate: the true effect is likely to be close to the estimate of the effect, but there is a possibility that it is substantially different.Low certainty: our confidence in the effect estimate is limited: the true effect may be substantially different from the estimate of the effect.Very low certainty: we have very little confidence in the effect estimate: the true effect is likely to be substantially different from the estimate of effect.

### 3.6 Adverse events

Ten trials (*n* = 640) reported adverse event data.^19^, ^29^, ^30^, ^31^, ^32^, ^35^, ^37^, ^38^, ^39^, ^45^ Only 1 trial reported the definition of SAE;^30^ no trial defined AE, device‐related AE, or AE leading to death (Supplementary Results S1).

**FIGURE 5:**
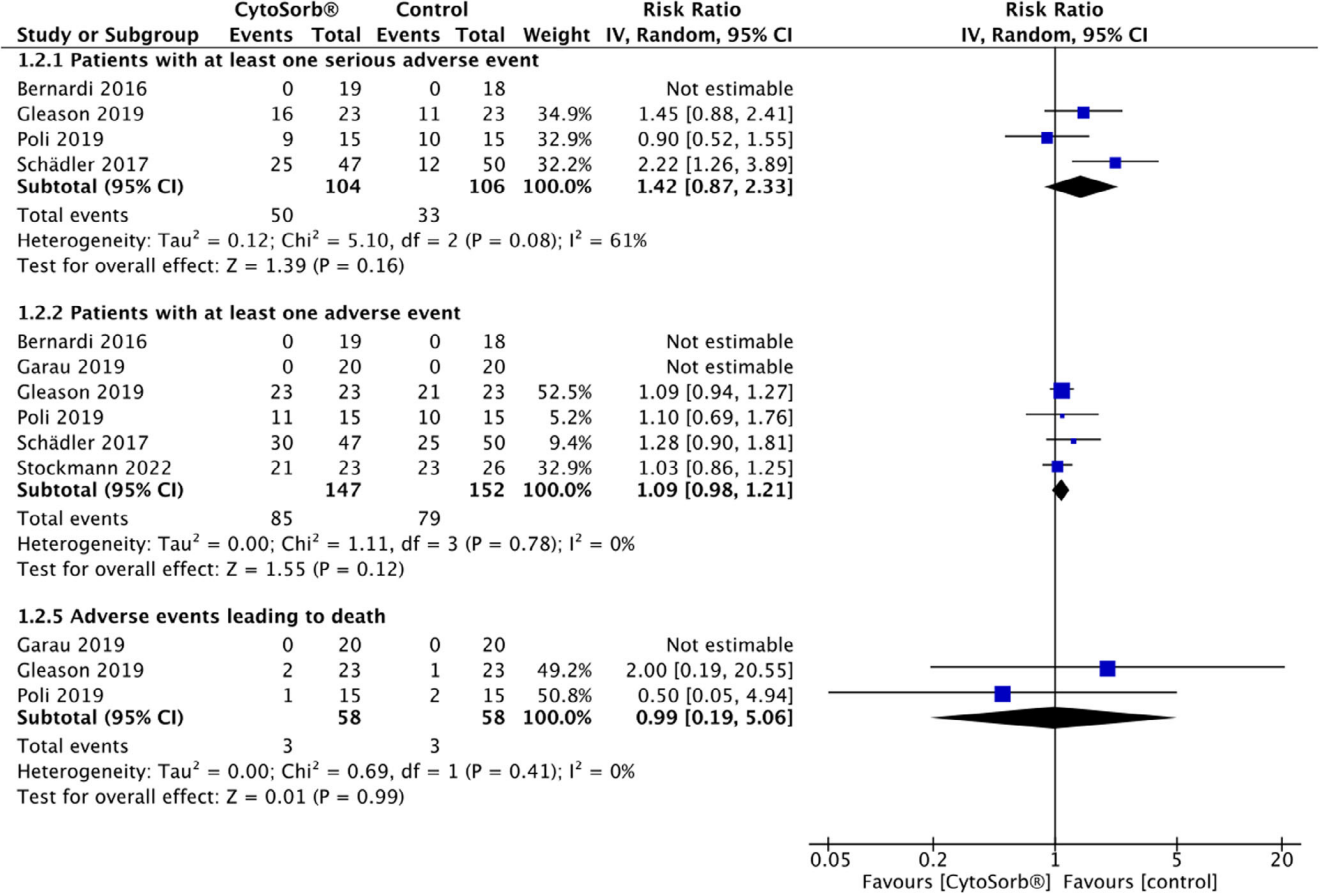
Forest plot of the relative risk of having at least one serious adverse event, one adverse event, or an adverse event leading to death

The number of patients experiencing at least 1 SAE was not significantly increased with hemoadsorption (4 trials, *n* = 210, 50 of 104 [48.08%] with CytoSorb® vs. 33 of 106 [31.13%] with control, RR = 1.42 [95% CI, 0.87–2.33], *p* = .16, *I* ^2^ = 61%, very low certainty), nor was the number of patients experiencing at least 1 AE (6 trials, *n* = 299, 85 of 147 [57.82%] with CytoSorb® vs. 79 of 152 [51.97%] with control, RR = 1.09 [95% CI, 0.98–1.21], *p* = .12, *I* ^2^ = 0%, very low certainty). The number of AE leading to death was reported by only 3 trials (*n* = 116) and did not significantly differ between groups (RR = 0.99 [95% CI, 0.19–5.06], *p* = .99, *I* ^2^ = 0%, very low certainty) (Figure 5). The TSA‐adjusted CI for AE was 0.88–1.35 (*D* ^2^ = 0%, RIS = 723), while the available information size was too small for performing a TSA for SAE (3.87%) and AE leading to death (0.58%).

The use of CytoSorb® was not associated with an increased number of SAEs (83 events in 85 patients with CytoSorb® vs. 70 of 88 with control, rate ratio = 1.18 [95% CI, 0.86–1.63], very low certainty) and AEs (623 events in 256 patients with CytoSorb® vs. 628 of 268 with control, rate ratio = 0.99 [95% CI, 0.86–1.15], very low certainty) ( Figure S4); 10 device‐related AEs were reported (7 trials, *n* = 290).

The quality of evidence for AE outcomes was very low, due to downgrading for very serious concerns of trial methodology (high risk of bias, lack of intention‐to‐treat analysis, lack of AE definition) and very serious imprecision. Adverse events related to hemoadsorption were underreported and not reported in a systematic manner. The definition of adverse events was unclear or not reported in most of the trials.

## 4 DISCUSSION

### 4.1 Key findings

We performed a comprehensive systematic review and meta‐analysis of mortality and adverse events of extracorporeal blood purification with the CytoSorb® device in critically ill patients with hyperinflammatory conditions. Using data from 16 randomized trials, we found an increased risk of mortality in the CytoSorb® treatment group at longest follow‐up available. Mortality at 30‐days or in‐hospital was also increased, with evidence from 11 trials. Attempting to control for random error using TSA yielded nonsignificant statistical results. Overall, the certainty of evidence was insufficient to draw firm conclusions on mortality effects and the safety profile of this hemoadsorption modality.

### 4.2 Relationship to previous studies

This is not the first study to suggest possible deleterious mortality effects of CytoSorb® therapy. The largest randomized trial performed so far on CytoSorb® use was published in 2017 and included data from 97 intubated patients with sepsis and acute lung injury or ARDS: it found no difference in the primary outcome (interleukin‐6 levels) but found a higher 60‐days mortality in patients on CytoSorb® (*p* = .039).^19^ The authors reported an adjusted analysis for patient morbidity and baseline imbalance in renal replacement therapy that supported no association of hemoperfusion with mortality (*p*‐value for effect shifted from 0.043 to 0.192). However, no information was available in the text or in the registered protocol regarding model adjustment.

Recently, the small randomized single‐center CYCOV trial assigned 34 patients with severe COVID‐19 pneumonia requiring venovenous ECMO to receive 72 h of Cytosorb® cytokine hemoadsorption or no treatment.^20^ The trial showed no significant difference in interleukin 6 concentrations (primary outcome) but found an increase in the 30‐day mortality in hemoadsorption patients. Baseline imbalance was present,^47^ but differences were sometimes in favor and other times against the intervention group. Various multiple regression and post‐hoc analyses were performed by the study authors, and those were unable to reveal any factor related to survival other than Cytosorb® treatment.

Other hemoadsorption extracorporeal modalities were also found to not be any better at reducing mortality than conventional therapy; examples of this are the EUPHRATES and ABDOMIX randomized trials examining the use of polymyxin B in sepsis.^4^, ^48^ These studies included 450 and 243 patients, respectively, and reported more frequent fatalities in the treatment group, although this was not statistically significant.

### 4.3 Significance of study findings

With absence of strong statistical support due to imprecision, the potential clinical significance of deleterious effects of hemoadsorption by CytoSorb® remains concerning. Uncertainty remains after TSA with regards to an assumed 20% relative risk increase in mortality associated with the device since only 35% of the required information size was reached, suggesting the need for further trials to improve the precision of estimates and allow firmer conclusions.

Some features of Cytosorb® and other hemoadsorption systems could theoretically explain the potential harm noticed in the treatment arms.

First, it is unclear whether nonselective removal of cytokines results in a beneficial or harmful imbalance. It may be that certain cytokines work together in beneficial ways and that the removal of one component may lead to a detrimental imbalance.

Second, the in vivo cytokine absorption capabilities of the device are unclear, since several randomized trial data available so far indicates no significant difference in most cytokines and pro‐inflammatory molecules in patients treated with CytoSorb® versus standard treatment.^19^, ^20^, ^30^, ^34^, ^35^, ^37^

Third, the device may adsorb various drugs, such as some antibiotics, antimycotics or antivirals.^49^, ^50^, ^51^, ^52^, ^53^ An in vitro study suggested the need for administration of an additional antibiotic dose within the first few hours of CytoSorb® treatment and for early therapeutic drug monitoring since all antimicrobial drugs tested were adsorbed by the cartridge in relevant amounts.^49^ However, reliable quantitative clinical data is needed to confirm these findings.^54^ Another in vitro study found that remdesivir and its main active metabolite were eliminated by CytoSorb®.^52^ Hence, without proper monitoring, some drugs may reach subtherapeutic levels and thus negatively impact patient outcome. This issue was also reported in other extracorporeal therapies.^55^, ^56^ The interactions between hemoadsorption therapies with effective COVID‐19 drugs such as dexamethasone and tocilizumab remain unknown. Similarly, effects on humoral antibody‐mediated immunity have not yet been investigated. CytoSorb® therapy was reported to be associated to a significant increase in analgesic requirements without impacting sedative requirements in a nonrandomized study, suggesting possible adsorption of opioids.^57^ However, removal of drugs can also be seen as an advantage. An example of such an application is emergency cardiac surgery in patients who received ticagrelor or rivaroxaban.^58^ Nonetheless, drug adsorption appears to be a side effect in the primarily intended context of cytokine removal and needs to be further studied through randomized trials.

Extracorporeal cytokine purification has been hypothesized to benefit patients with hyperinflammatory syndromes: these included some conditions as severe sepsis, vasoplegic shock, ARDS, burns, pancreatitis, liver failure, or complex cardiac surgery. The capacity of extracorporeal blood purification devices to decrease inflammation is unclear. The present study included only trials including hyperinflammatory conditions (e.g., complex cardiac surgery with long CPB time, severe sepsis). Given the issue of a small cumulative sample size, we failed to find an improvement in mortality in any clinical setting. Whether hemoadsorption with CytoSorb® could benefit some specific disease phenotypes remains unclear.

Adverse events and safety outcomes were underreported and not systematically reported, and poorly defined ‐or even undefined‐ when they were. The lack of an intention‐to‐treat analysis in some studies could increase the risk of missing adverse events, whether or not those were related to hemoadsorption. CytoSorb® hemoadsorption consists in integrating the cartridge device into an extracorporeal circuit linked to patients through a central venous access. Interactions with blood components were reported by some randomized trials. One trial performed in cardiac surgery reported that a mean drop of 56% in platelets was observed after the initiation of CytoSorb® treatment during CPB (vs. a 4% drop in control); platelets returned to pre‐treatment levels after the end of CytoSorb® treatment in “most cases.”^32^ Another cardiac surgery trial found a significant lower factor II and XII activity.^30^ A study performed in sepsis found lower levels of platelets, white blood cells, albumin, and total protein in the CytoSorb® group.^19^ Extracorporeal blood circulation is associated with some well‐known adverse effects and complications that could easily be systematically assessed and reported.

We found that half of the published trials were financially supported by the CytoSorb® manufacturer and that more than half of published trials had at least one author with some kind of conflict of interest. We found that only two trials clearly stated that no industry funding or financial conflict of interest were present. These findings are not surprising since CytoSorb® therapy is a relatively expensive experimental therapy and industry support is not rare in the field of extracorporeal therapy.^5^ A Cochrane review found that sponsorship of drug and device studies by the manufacturing company leads to more favorable efficacy results and conclusions than sponsorship by other sources in primary research studies.^59^ However, a large meta‐epidemiological study found that industry‐funded RCTs are reported to be the minority in intensive care medicine.^60^ The same study found no evidence that industry‐funded trials yielded more favorable results or were less likely to reach unfavorable conclusions.^60^ Non‐financial conflicts of interest were also frequent; whether this is associated to biased results remains uninvestigated.^25^ Our subgroup analyses with financial or non‐financial conflict of interest stratification were inconclusive.

### 4.4 Strengths and limitations of the study

The present systematic review followed a pre‐published protocol and the Cochrane methodology, using TSA to assess the risk of type 1 and 2 errors and the GRADE approach for summarizing the certainty of the evidence. The aim of this review was to provide useful and exploratory information on mortality effects and safety of blood purification using CytoSorb®. This use of this device is approved in Europe and USA for some specific indications and thousands of patients received CytoSorb® therapy so far, but no randomized trial was performed specifically to show safety and major clinical improvement. Our study could partially fill this gap, even if it has various limitations mainly related to the quantity and quality of randomized trials. Most of the RCTs were single‐center; few trials were at low risk of bias and some trials lacked an intention‐to‐treat analysis. All eligible RCTs had a surrogate outcome as primary outcome, mainly related to cytokine and inflammatory markers after treatment, and were not adequately powered for mortality. The small size of each trial could increase the risk of baseline imbalance, an issue that was reported by one trial.^19^

Adverse event definition was lacking in most of the trials and those were not systematically reported, as was the case for safety outcomes. These limitations in safety and AE outcomes increase the risk of false negative results (i.e., a difference in safety and AE actually exists) and limit the external validity of the results. To increase power and fully assess safety, we included trials from various settings. A possible subgroup effect cannot be excluded and various exploratory subgroup analyses are presented. These subgroup analyses were done exploratively, without adjustment for multiplicity. No adjustment was made to account for multiplicity of the secondary outcomes and subgroup analyses increasing the risk of false positive findings, since this issue remains unresolved in the context of meta‐analysis. To decrease the risk of publication bias, we decided to contact the corresponding authors of trials listed in trial registers for unpublished data. However, several corresponding authors did not reply or did not share any data.

## 5 CONCLUSION

Low certainty of evidence showed that the use of CytoSorb® might increase mortality in critically ill patients. Adverse events did not differ between groups, but they were underreported and not systematically evaluated. Industry funding and conflicts of interest were frequent. Considerable uncertainty about the findings do not allow firm conclusions and suggest the need of further high‐quality randomized trials before systematic use of CytoSorb® hemoadsorption. Adverse events and mortality must be systematically assessed.

## AUTHOR CONTRIBUTIONS

Conceptualization: Raoul Schorer, Alessandro Putzu. Literature search: Marc Heymann, Alessandro Putzu. Hits screened and reviewed: Marc Heymann, Alessandro Putzu. Data curation: Marc Heymann, Alessandro Putzu. Analysis of data: Alessandro Putzu. Supervision: Raoul Schorer, Alessandro Putzu. Access to data: all authors. Interpretation of data: all authors. Manuscript drafting: Marc Heymann, Alessandro Putzu. Manuscript revision, editing, and approval: all authors.

## Supporting information


**Appendix S1:** Supporting Information

## Data Availability

Template data collection forms; data extracted from included studies; data used for all analyses; analytic code; any other materials used in the review: available from the corresponding authors on reasonable request.
