# Supplementary material for: Mortality and adverse events of hemoadsorption with CytoSorb® in critically ill patients: A systematic review and meta‐analysis of randomized controlled trials
Source: Acta Anaesthesiol Scand. 2022 Jul 18;66(9):1037–50. doi: 10.1111/aas.14115 (PMC9541789; doi:10.1111/aas.14115)
Supplement: Supplementary file 1 — Appendix S1: Supporting Information [file AAS-66-1037-s001.docx]

**SUPPLEMENTARY MATERIAL**

**Title**

Mortality and adverse events of hemoadsorption with CytoSorb® in critically ill patients: a systematic review and meta-analysis of randomized controlled trials

**Authors**

Marc Heymann, MD, MPH; Raoul Schorer, MD; Alessandro Putzu, MD*

Division of Anesthesiology, Department of Acute Medicine, Geneva University Hospitals, Geneva, Switzerland

*Corresponding author: Alessandro.Putzu@hcuge.ch.

Table of contents

[**Supplementary Table S1 - PRISMA 2020 Checklist** 4](#_Toc108594747)

[**Supplementary Methods S1 – Search strategies** 7](#_Toc108594748)

[**Supplementary Methods S2 – Protocol amendments.** 8](#_Toc108594749)

[**Supplementary Table S2 – Major exclusions with reasons** 8](#_Toc108594750)

[**Supplementary Table S3 – Further characteristics of included trials** 9](#_Toc108594751)

[**Supplementary Figure S1 - Risk of bias summary for adverse events: review authors' judgements about each risk of bias item for each included study.** 11](#_Toc108594752)

[**Supplementary Table S4 – Details on financial and non-financial conflict of interest** 12](#_Toc108594753)

[**Supplementary Table S5 – Primary outcome: subgroup and sensitivity analyses for mortality** 15](#_Toc108594754)

[**Supplementary Figure S2 – Primary outcome: subgroup analysis on mortality at longest follow-up available according to setting** 16](#_Toc108594755)

[**Supplementary Figure S3 – Primary outcome: exploratory subgroup analyses on mortality at longest follow-up available according to different medical conditions.** 17](#_Toc108594756)

[**Supplementary Figure S4 – Primary outcome: funnel plot.** 18](#_Toc108594758)

[**Supplementary Table S6 – Certainty of the body of evidence assessment using the grading of recommendations assessment, development and evaluation (GRADE) framework: primary and secondary outcomes.** 19](#_Toc108594760)

[**Supplementary Results S1 – Adverse events reporting.** 21](#_Toc108594761)

[**Supplementary Figure S5 – Secondary outcomes: number of adverse events** 21](#_Toc108594762)

# **Supplementary Table S1 - PRISMA 2020 Checklist**

*Abstract*

| **Section and Topic** | **Item #** | **Checklist item** | **Reported (Yes/No)** |
| --- | --- | --- | --- |
| **TITLE** | | |  |
| Title | 1 | Identify the report as a systematic review. | Reported |
| **BACKGROUND** | | |  |
| Objectives | 2 | Provide an explicit statement of the main objective(s) or question(s) the review addresses. | Reported |
| **METHODS** | | |  |
| Eligibility criteria | 3 | Specify the inclusion and exclusion criteria for the review. | Partially reported (words limit) |
| Information sources | 4 | Specify the information sources (e.g. databases, registers) used to identify studies and the date when each was last searched. | Partially reported (words limit) |
| Risk of bias | 5 | Specify the methods used to assess risk of bias in the included studies. | Not reported (words limit) |
| Synthesis of results | 6 | Specify the methods used to present and synthesise results. | Reported |
| **RESULTS** | | |  |
| Included studies | 7 | Give the total number of included studies and participants and summarise relevant characteristics of studies. | Reported |
| Synthesis of results | 8 | Present results for main outcomes, preferably indicating the number of included studies and participants for each. If meta-analysis was done, report the summary estimate and confidence/credible interval. If comparing groups, indicate the direction of the effect (i.e. which group is favoured). | Reported |
| **DISCUSSION** | | |  |
| Limitations of evidence | 9 | Provide a brief summary of the limitations of the evidence included in the review (e.g. study risk of bias, inconsistency and imprecision). | Reported |
| Interpretation | 10 | Provide a general interpretation of the results and important implications. | Reported |
| **OTHER** | | |  |
| Funding | 11 | Specify the primary source of funding for the review. | Not reported (words limit) |
| Registration | 12 | Provide the register name and registration number. | Not reported (words limit) |

*Manuscript*

| **Section and Topic** | **Item #** | **Checklist item** | **Location where item is reported** |
| --- | --- | --- | --- |
| **TITLE** | | |  |
| Title | 1 | Identify the report as a systematic review. | 1 |
| **ABSTRACT** | | |  |
| Abstract | 2 | See the PRISMA 2020 for Abstracts checklist. | See above |
| **INTRODUCTION** | | |  |
| Rationale | 3 | Describe the rationale for the review in the context of existing knowledge. | 4 |
| Objectives | 4 | Provide an explicit statement of the objective(s) or question(s) the review addresses. | 5 |
| **METHODS** | | |  |
| Eligibility criteria | 5 | Specify the inclusion and exclusion criteria for the review and how studies were grouped for the syntheses. | 6 |
| Information sources | 6 | Specify all databases, registers, websites, organisations, reference lists and other sources searched or consulted to identify studies. Specify the date when each source was last searched or consulted. | 5 |
| Search strategy | 7 | Present the full search strategies for all databases, registers and websites, including any filters and limits used. | Suppl. |
| Selection process | 8 | Specify the methods used to decide whether a study met the inclusion criteria of the review, including how many reviewers screened each record and each report retrieved, whether they worked independently, and if applicable, details of automation tools used in the process. | 6 |
| Data collection process | 9 | Specify the methods used to collect data from reports, including how many reviewers collected data from each report, whether they worked independently, any processes for obtaining or confirming data from study investigators, and if applicable, details of automation tools used in the process. | 6 |
| Data items | 10a | List and define all outcomes for which data were sought. Specify whether all results that were compatible with each outcome domain in each study were sought (e.g. for all measures, time points, analyses), and if not, the methods used to decide which results to collect. | 6,7 |
|  | 10b | List and define all other variables for which data were sought (e.g. participant and intervention characteristics, funding sources). Describe any assumptions made about any missing or unclear information. | 6,7 |
| Study risk of bias assessment | 11 | Specify the methods used to assess risk of bias in the included studies, including details of the tool(s) used, how many reviewers assessed each study and whether they worked independently, and if applicable, details of automation tools used in the process. | 7 |
| Effect measures | 12 | Specify for each outcome the effect measure(s) (e.g. risk ratio, mean difference) used in the synthesis or presentation of results. | 8 |
| Synthesis methods | 13a | Describe the processes used to decide which studies were eligible for each synthesis (e.g. tabulating the study intervention characteristics and comparing against the planned groups for each synthesis (item #5)). | 8,9 |
|  | 13b | Describe any methods required to prepare the data for presentation or synthesis, such as handling of missing summary statistics, or data conversions. | 8,9 |
|  | 13c | Describe any methods used to tabulate or visually display results of individual studies and syntheses. | 8 |
|  | 13d | Describe any methods used to synthesize results and provide a rationale for the choice(s). If meta-analysis was performed, describe the model(s), method(s) to identify the presence and extent of statistical heterogeneity, and software package(s) used. | 8,9 |
|  | 13e | Describe any methods used to explore possible causes of heterogeneity among study results (e.g. subgroup analysis, meta-regression). | 8,9 |
|  | 13f | Describe any sensitivity analyses conducted to assess robustness of the synthesized results. | 8,9 |
| Reporting bias assessment | 14 | Describe any methods used to assess risk of bias due to missing results in a synthesis (arising from reporting biases). | 8 |
| Certainty assessment | 15 | Describe any methods used to assess certainty (or confidence) in the body of evidence for an outcome. | 9 |
| **RESULTS** | | |  |
| Study selection | 16a | Describe the results of the search and selection process, from the number of records identified in the search to the number of studies included in the review, ideally using a flow diagram. | 9 |
|  | 16b | Cite studies that might appear to meet the inclusion criteria, but which were excluded, and explain why they were excluded. | Suppl. |
| Study characteristics | 17 | Cite each included study and present its characteristics. | 9,10,table |
| Risk of bias in studies | 18 | Present assessments of risk of bias for each included study. | 10, suppl. |
| Results of individual studies | 19 | For all outcomes, present, for each study: (a) summary statistics for each group (where appropriate) and (b) an effect estimate and its precision (e.g. confidence/credible interval), ideally using structured tables or plots. | Figures, suppl. |
| Results of syntheses | 20a | For each synthesis, briefly summarise the characteristics and risk of bias among contributing studies. | 10 |
|  | 20b | Present results of all statistical syntheses conducted. If meta-analysis was done, present for each the summary estimate and its precision (e.g. confidence/credible interval) and measures of statistical heterogeneity. If comparing groups, describe the direction of the effect. | 11-14 |
|  | 20c | Present results of all investigations of possible causes of heterogeneity among study results. | 11-14, suppl |
|  | 20d | Present results of all sensitivity analyses conducted to assess the robustness of the synthesized results. | 11-14, suppl |
| Reporting biases | 21 | Present assessments of risk of bias due to missing results (arising from reporting biases) for each synthesis assessed. | 10 |
| Certainty of evidence | 22 | Present assessments of certainty (or confidence) in the body of evidence for each outcome assessed. | 11-14, suppl |
| **DISCUSSION** | | |  |
| Discussion | 23a | Provide a general interpretation of the results in the context of other evidence. | 15-18 |
|  | 23b | Discuss any limitations of the evidence included in the review. | 15-18 |
|  | 23c | Discuss any limitations of the review processes used. | 18 |
|  | 23d | Discuss implications of the results for practice, policy, and future research. | 15-19 |
| **OTHER INFORMATION** | | |  |
| Registration and protocol | 24a | Provide registration information for the review, including register name and registration number, or state that the review was not registered. | 5 |
|  | 24b | Indicate where the review protocol can be accessed, or state that a protocol was not prepared. | 5 |
|  | 24c | Describe and explain any amendments to information provided at registration or in the protocol. | Methods |
| Support | 25 | Describe sources of financial or non-financial support for the review, and the role of the funders or sponsors in the review. | 5 |
| Competing interests | 26 | Declare any competing interests of review authors. | 5 |
| Availability of data, code and other materials | 27 | Report which of the following are publicly available and where they can be found: template data collection forms; data extracted from included studies; data used for all analyses; analytic code; any other materials used in the review. | 3 |

*From:*  Page MJ, McKenzie JE, Bossuyt PM, Boutron I, Hoffmann TC, Mulrow CD, et al. The PRISMA 2020 statement: an updated guideline for reporting systematic reviews. BMJ 2021;372:n71. doi: 10.1136/bmj.n71

For more information, visit: <http://www.prisma-statement.org/>

# **Supplementary Methods S1 – Search strategies**

*PubMed*

(haemoperfusion[tiab] OR hemoperfusion[tiab] OR hemoadsorption[tiab] OR hemadsorption[tiab] OR haemoadsorption[tiab] OR haemadsorption[tiab] OR (cytokine*[tiab] AND adsorption*[tiab]) OR cytosorb[tiab]) AND ("randomized Controlled Trial"[Publication Type] OR "Controlled Clinical Trial"[Publication Type] OR "Clinical Trials as Topic"[Mesh:NoExp] OR randomi*[tiab] OR randomly[tiab] OR placebo[tiab] OR “case control”[tiab] OR blind*[tiab] OR “clinical trial”[tiab] OR “clinical trials”[tiab])

*EMBASE*

(haemoperfusion:ab,ti OR hemoperfusion:ab,ti OR hemoadsorption:ab,ti OR hemadsorption:ab,ti OR haemoadsorption:ab,ti OR haemadsorption:ab,ti OR (cytokine*:ab,ti AND adsorption*:ab,ti) OR cytosorb:ab,ti) AND ('randomised controlled trial'/exp OR 'controlled clinical trial'/exp OR randomi*:ab,ti OR randomly:ab,ti OR placebo:ab,ti OR ‘case control’:ab,ti OR blind*:ab,ti OR ‘clinical trial’:ab,ti OR ‘clinical trials’:ab,ti)

*CENTRAL*

#1: (haemoperfusion OR hemoperfusion OR hemadsorption OR hemoadsorption OR haemadsorption OR haemoadsorption OR (cytokine* AND adsorption*) OR cytosorb):ti,ab

#2: (random* OR randomly OR placebo OR “case control” OR blind* OR “clinical trial” OR “clinical trials”):ti,ab

#3: #1 AND #2

*Trial Registry: ClinicalTrials.gov*

https://clinicaltrials.gov/

Advanced Search >

Study Type: interventional studies (Clinical Trials)

Study Results: All Studies

Interventions: hemadsorption OR hemoadsorption OR haemadsorption OR haemoadsorption OR CytoSorb OR “cytokine adsorption” OR “cytokine removal”

*Trial Registry: World Health Organization, International Clinical Trials Registry Platform (https://trialsearch.who.int/Default.aspx)*

Standard search in Title, Primary sponsor, Health Condition(s), Intervention(s), Countries of recruitment, Main ID, Secondary ID(s): hemadsorption OR hemoadsorption OR haemadsorption OR haemoadsorption OR CytoSorb OR “cytokine adsorption” OR “cytokine removal”

Phases: All

# **Supplementary Methods S2 – Protocol amendments.**

| **Nature of the Deviation** | **Description of the Deviation** | **Reason for the Deviation** |
| --- | --- | --- |
| Inclusion of unpublished trials | The original protocol did not plan to include data from unpublished trials. | Review authors decided to include unpublished trials to decrease the risk of publication bias. |
| Trial sequential analysis | The original protocol reported a RRI of 50% in mortality at longest follow-up, that was drawn from one of the largest RCT on the use of CytoSorb® (Schädler D et al, 2017 PLoS One 12:e0187015) and therefore considered clinicallly plausible. | During a peer review process, a RRI of 50% was judged to be clinically implausible. Consequently, a RRI of 20% was assumed for the primary analysis. |
| Trial sequential analysis | The original protocol planned a TSA only for the primary outcome | During a peer review process, it was judged to be of interest to perform a TSA for primary and secondary outcome. It was not possible to perform a TSA for secondary outcomes with rate ratio as effect estimate. |
| Non-financial conflicts of interest | The original protocol reported the assessment of only financial conflicts of interest. | During a peer review process, it was evaluated that the assessment of non-financial conflicts of interest would be of interest. |

RRI, relative risk increase; TSA, trial sequential analysis

# **Supplementary Table S2 – Major exclusions with reasons**

| **Trial** | **Format** | **DOI/reference** | **Reason for exclusion** |
| --- | --- | --- | --- |
| Bernardi 2019 | Full-text | ASAIO J (2019) 19;65(7) | Overlap population |
| Deppe 2018 | Abstract | Thorac Cardiovasc Surg (2018) 66(Suppl 01) | Non-randomized study |
| Iskender 2017 | Full-text | J Heart Lung Transplant (2017) 20(17) | Animal study |
| Kellum 2008 | Full-text | Crit Care Med (2008) 36:1 | Lack of control group |
| László 2017 | Abstract | Crit Care (2017) 21(Suppl 1):58 | Overlap population |
| Laubach 2018 | Abstract | Intensive Care Med Exp (2018) 6(Suppl 2):40 | Lack of control group |
| Liu 2021 | Full-text | Eur Rev Med Pharmacol Sci (2021) 25: 2403-2408 | Use of another device |
| Poli 2018 | Abstract | Critical Care (2018) 22(Suppl 1):82 | Overlap population |
| Rampino 2020 | Full-text | Blood Purif (2020) 12;1 | Non-randomized study |
| Schädler 2013 | Abstract | Critical Care (2013) 17(Suppl 2) | Overlap population |
| Schädler 2013 | Abstract | Am J Respir Cit Care Med (2013)187: A5241 | Overlap population |
| Scharf 2021 | Full-text | Crit Care (2021) 25:41 | Non-randomized study |
| Taleska Stupica 2020 | Abstract | J Cardiothorac Vasc Anesth (2020) 34(Suppl 1) | Overlap population |
| Vinay Kumar 2020 (1) | Abstract | Indian J Gastroenterol (2020) 39 (Suppl 1):S1–S141 | Use of another device |
| Vinay kumar 2020 (2) | Abstract | Hepatology (2020) 72 (suppl 1) | Use of another device |
| Wisgrill 2020 | Full-text | J Transl Med (2020) 18:202 | Overlap population |

# **Supplementary Table S3 – Further characteristics of included trials**

| **Trial** | **Primary outcome** | **Sample size calculation for primary outcome** | **Age CytoSorb®** | **Age control** | **Female CytoSorb®** | **Female control** | **Severity score CytoSorb®** | **Severity score control** | **Other mortality follow-up** |
| --- | --- | --- | --- | --- | --- | --- | --- | --- | --- |
| *Published trials* | | | | | | | | |  |
| Asch 2021 | Selected cytokine and infection parameters levels | NR | 65 (53-70) | 69 (56-81) | 3 | 1 | Euroscore II 8.5 (2.7-16.4) | Euroscore II 3.6 (2.6-11.8) | - |
| Bernardi 2016 | Selected cytokine levels | Yes | 64 (30–81) | 69 (51–81) | 7 | 4 | Euroscore 4.0 ± 3.6 | Euroscore 6.0 ± 4.6 | - |
| Diab 2022 | Change in SOFA score | Yes | 68.5 (58-76) | 69 (60-76) | 40 | 33 | Euroscore II 19.1 ± 17.3 | Euroscore II 20.2 ± 17.8 | - |
| Garau 2019 | Selected cytokine levels | Yes | 67.9 ± 12.7 | 72.7 ± 9.2 |  |  | Euroscore II 6.1 ± 2.2 | Euroscore II 6.3 ± 2.9 | - |
| Gleason 2019 | Plasma free haemoglobin levels | NR | 66 ± 8 | 61 ± 17 | 10 | 5 | NR | NR | - |
| Hawchar 2019 | Unclear | Not performed | 60 ± 10 | 71 ± 14 | 3 | 4 | APACHE II 26 ± 9 | APACHE 30±6 | 2-days |
| Holmén 2022 | Amount of norepinephrine | Yes | 72 (62-74) | 70 (64-76) | 1 | 1 | NR | NR | - |
| Poli 2019 | Selected cytokine levels | Yes | 67 (44–76) | 69 (49–80) | 2 | 4 | Euroscore II 3.0 (2.2–9.1) | Euroscore II 5.1 (2.1–7.2) | ICU |
| Schädler 2017 | Interleukin-6 levels | Not performed | 66.0 (55–73) | 65 (56.5–71) | 12 | 15 | APACHE II score 24.6 ± 5.2 | APACHE II score 23.8 ± 5.7 | 7-days, 28-days |
| Stockmann 2022 | Time until resolution of vasoplegic shock | Yes, but planned 100 patients | 61 (58-65) | 66 (60-71) | 2 | 6 | SOFA score 14 (13–15) | SOFA score 14 (13–16) | 7-days, 30-days |
| Supady 2021 | Interleukin-6 levels | Yes | 62.0 (54.0–71.5) | 59.0 (43.5–66.5) | 5 | 4 | SOFA 9.0 (8.0–10.0) | SOFA 9.0 (7.0–10.5) | 30-days |
| Supady 2022 | Interleukin-6 levels | Yes | 60.5 (50.8–69.8) | 64.0 (54.0–71.0) | 7 | 7 | APACHE II score 31 (27–33) | APACHE II score 34 (26–38) | ICU mortality |
| Taleska Stupica 2020 | Selected cytokine and infection parameters levels | Yes | 70.5 (34–80) | 71 (31–85) | 6 | 6 | Euroscore II 2.26 (0.96–10.43) | Euroscore II 2.78 (0.96–10.74) | 30-days |
| Wagner 2019 | Selected myocardial, monocyte and vascular miRNAs plasma levels | NR | 50 ± 10 | 54 ± 15 | 2 | 0 | Euroscore II. 4.2 ± 2.1 | Euroscore II 4.1 ± 1.9 | - |
| *Unpublished trials* | | | | | | | | |  |
| NCT03145441 | The incidence of early rejection and cytokine and complement levels | NR | NR | NR | NR | NR | NR | NR | - |
| NCT03523039 | Selected cytokine levels and rate of intervention-related complications | NR | NR | NR | NR | NR | NR | NR | - |
| NCT04361526 | Mechanical ventilation-free days | NR | NR | NR | NR | NR | NR | NR | - |
| NCT04518969 | Selected cytokine levels | NR | NR | NR | NR | NR | NR | NR | - |

Data reported as mean ± standard deviation or median (interquartile range). NR, not reported

# **Supplementary Figure S1 - Risk of bias summary for adverse events: review authors' judgements about each risk of bias item for each included study.**


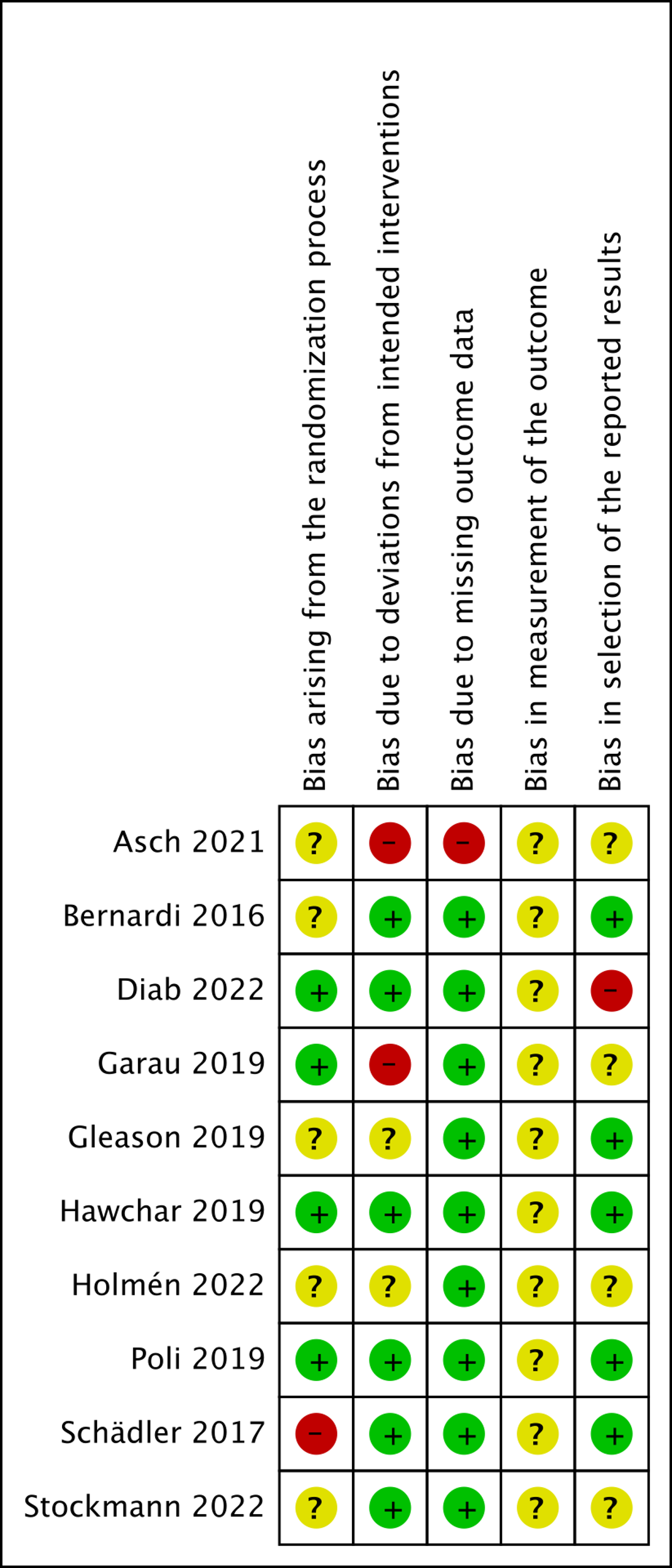


# **Supplementary Table S4 – Details on financial and non-financial conflict of interest**

| **Trial** | **Industrial funding?** | **Funding details** | **Role of funder (design, conduct, analysis, reporting)** | **Author with a financial conflict of interest?** | **Details on authors conflict of interest** | **Stage of trial contribution (design, conduct, analysis, reporting)** | **Overall judgment about conflict of interest** | **Author with a non-financial conflict of interest** |
| --- | --- | --- | --- | --- | --- | --- | --- | --- |
| *Published trials* | | | | | | | |  |
| Asch 2021 | NR | NR | NR | No | - | - | Unclear concerns | No |
| Bernardi 2016 | Yes | “Materials for this study have been partly financially supported by CytoSorbents Europe GmbH. All other sources of funding for laboratory measurements and human resources were departmental and institutional funding.” | NR | Yes | 2 of 11 authors (the first and second-last author) received travel funding for a lecture from CytoSorbents Europe GmbH. | First author: design, conduct, reporting; second-last author: design, reporting | Notable concern | Yes: 2 authors acknowledged experts in the field, with multiple publications |
| Diab 2022 | Yes | “Additional funding and hemoadsorbers were provided by CytoSorbents Europe GmbH” | “The study was designed, conducted, analyzed, and interpreted by the investigators, completely independent of all funding sources” | Yes | 4 of 33 authors had various type of relation with CytoSorbents Europe GmbH. | Second-last author: design, conduct, analysis, reporting; 31^st^ author: design, conduct, reporting; Fifth author: design, analysis, reporting; Sixth author: conduct. | Notable concern | No |
| Garau 2019 | Yes | “Unrestricted grant from CytoSorbents Europe GmbH.” | NR | No | - | - | Notable concern | No |
| Gleason 2019 | Yes | “CytoSorbents Corporation was the sponsor of this pilot study and reimbursed patient study-related expenses to the institutions involved.” | “The original manuscript was drafted by the principal investigators from each site, independently of the Company. However, as typically specified in clinical trial agreements for such studies, the company reserved and exercised the right to review any manuscripts or presentations for factual errors prior to submission.” | Yes | 1 of 10 authors. Last, corresponding author: served as a member of the Cytosorb Cardiac Advisory Board (without compensation). | NR | Notable concern | No |
| Hawchar 2019 | No | Supported by various research grants | - | Yes | 1 of 6 authors. Last, corresponding author: received honoraria for lecturing and consulting for CytoSorbents. | Last, corresponding author: all roles | Notable concern | Yes: 2 authors with multiple publications; 1 author acknowledged expert in the field |
| Holmén 2022 | Yes | “The cytokine hemadsorption cartridges were supplied by CytoSorbents Europe GmbH free of charge” | “The company had no impact on study design, writing or interpreting the data, or decision to submit” | No | - | - | Notable concern | No |
| Poli 2019 | Yes | “Partially supported by CytoSorbents Europe GmbH.” | The company was allowed to read the draft manuscript before submission but had no influence on its content or decision for submission. | Yes | 1 of 9 authors. The last, corresponding author has received speaker honoraria from Cytosorbent SA. | Last author: all stages | Notable concern | Yes: 1 author acknowledged expert in the field, with multiple publications |
| Schädler 2017 | Yes | “The study was supported by Cytosorbents Corporation, New Jersey, United States.” | “Cytosorbents was involved in study design and data collection. Cytosorbents had no role in analysis, decision to publish or preparation of the manuscript. Cytosorbents does not employ and does not pay consultancies to any of the authors” | Yes | 3 of 15 authors. Last author: received a research grant for study administration and coordination by Cytosorbents. Seventh author: received honorary from Cytosorbents. First, corresponding author and last author: received refunds of travelling expenses by Cytosorbents. | First, corresponding author: conduct, reporting. Last author: design, conduct, reporting. Seventh author: conduct. | Notable concern | Yes: 2 authors acknowledged experts in the field, with multiple publications |
| Stockmann 2022 | No | No external funding | - | Yes | 2 of 17 authors. 8th author: received research funding and honoraria for workshops and lectures from Cytosorbents, outside the submitted work; 7th author: received grants from Cytosorbents, outside the submitted work. | NR | Notable concern | Yes: 1 author acknowledged experts in the field, with multiple publications |
| Supady 2021 | No | “There was no funding source for this study” | - | Yes | 5 of 23 authors. First, corresponding author: received research grants and lecture fees from CytoSorbents, outside of the published work. Third author: received financial support from CytoSorbents for attending a scientific meeting. 11th author: received honoraria from CytoSorbents for a presentation during a scientific workshop. 10th author: received lecture honoraria from CytoSorbents. Last author: received research grants, lecture fees and travel support from CytoSorbents, all outside of the published work. | First, corresponding author: design, analysis, reporting. Third author: conduct, analysis. 11th author: conduct, analysis. 10th author: conduct, analysis. Last author: conduct, analysis, reporting | Notable concern | Yes: 1 author acknowledged expert in the field, with multiple publications |
| Supady 2022 | Yes | Additional funding was provided by CytoSorbents Europe GmbH. | Unclear. “CytoSorbents Europe GmbH may commercially exploit the research results from this study” | Yes | 4 of 12 authors. First, corresponding author: research grants and lecture fees from CytoSorbents, outside the submitted work. Fourth author: honoraria from CytoSorbents for a presentation during a scientific workshop. Fifth author: lecture honoraria from CytoSorbents. Last author: research grants, lecture fees, and travel support from CytoSorbents, all outside the submitted work. | First, corresponding author: design, conduct, analysis, reporting. Fourth author: conduct, analysis. Fifth author: conduct, analysis. Last author: design, conduct, analysis, reporting | Notable concern | Yes: 1 author acknowledged expert in the field, with multiple publications |
| Taleska Stupica 2020 | No | This work was supported by the Slovenian Research Agency and by the University Medical Centre Ljubljana | - | No | - | - | No notable concern | No |
| Wagner 2019 | No | This work was supported by an institutional grant | - | No | - | - | No notable concern | No |
| *Unpublished trials* | | | | | | | |  |
| NCT03145441 | NR | - | - | NR | - | - | Unclear concerns | Yes: 1 author with multiple publications |
| NCT03523039 | NR | - | - | NR | 1 author had conflicts of interest in another published study (Poli et al. Critical Care 2019 23:108) | - | Notable concern about conflict of interest | Yes: 1 author acknowledged expert in the field, with multiple publications |
| NCT04361526 | NR | - | - | NR | - | - | Unclear concerns | No |
| NCT04518969 | NR | - | - | NR | - | - | Unclear concerns | Yes: 1 author acknowledged expert in the field, with multiple publications |

NR, not reported

# **Supplementary Table S5 – Primary outcome: subgroup and sensitivity analyses for mortality**

| **Analysis** | **Effect estimate** | **p value for interaction** |
| --- | --- | --- |
| Primary analysis | RR=1.24 [95% CI, 1.04 to 1.49] | - |
| Sensitivity analysis: fixed effects model | RR=1.24 [95% CI, 1.04 to 1.49] | - |
| Sensitivity analysis: risk difference | Risk difference=0.04 [95% CI -0.01 to 0.09] | - |
| Sensitivity analysis:  - published trials (longest follow-up)  - published trials (30-days) | RR=1.27 [95% CI, 1.03 to 1.57]  RR=1.43 [95% CI, 1.07 to 1.91] | - |
| Sensitivity analysis: TSA published trials | RR=1.24 [TSA-adjusted CI, 0.80 to 2.03], D2=19%, RIS=2786 | - |
| Subgroup analysis:  - published trials  - unpublished trials | RR=1.27 [95% CI, 1.03 to 1.57]  RR=1.14 [95% CI, 0.65 to 2.00] | 0.71 |
| Subgroup analysis: risk of bias  - low risk of bias  - some concerns/high risk of bias | RR= 1.40 [95% CI, 0.86 to 2.25]  RR=1.19 [95% CI, 0.93 to 1.52] | 0.56 |
| Subgroup analysis: financial conflict of interest  - Some concerns/unclear concerns  - No concerns | RR=1.28 [95% CI, 0.99, 1.65]  Not estimable (0 fatal events) | Not estimable |
| Subgroup analysis: non-financial conflict of interest  - Some concerns/unclear concerns  - No concerns | RR=1.48 [95% CI, 1.08 to 2.02]  RR=1.08 [95% CI, 0.79 to 1.48] | 0.17 |

CI, confidence interval; RR, relative risk; TSA, trial sequential analysis.

# **Supplementary Figure S2 – Primary outcome: subgroup analysis on mortality at longest follow-up available according to setting**

**
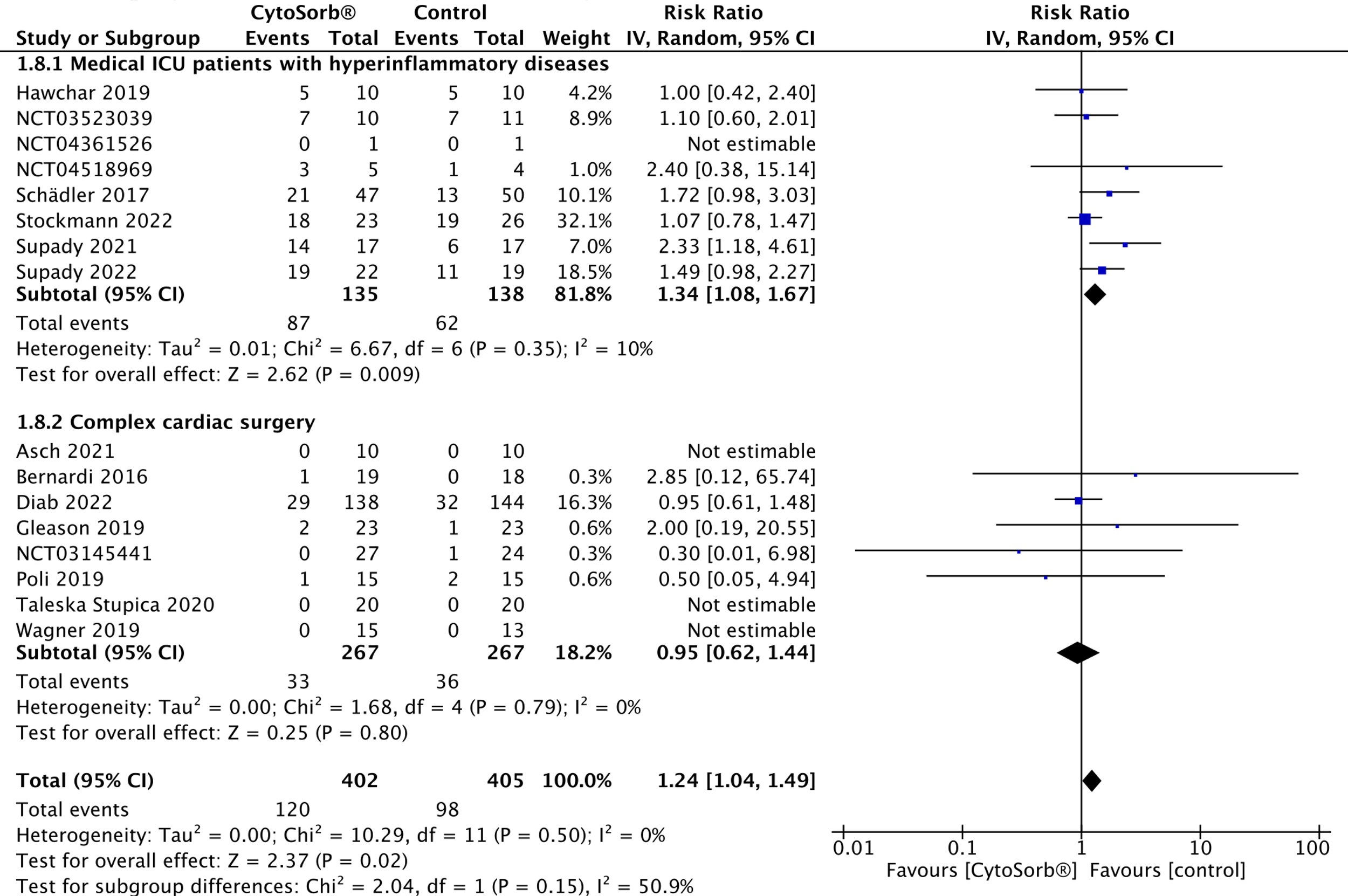
**

# **Supplementary Figure S3 – Primary outcome: exploratory subgroup analyses on mortality at longest follow-up available according to different medical conditions.**

# **
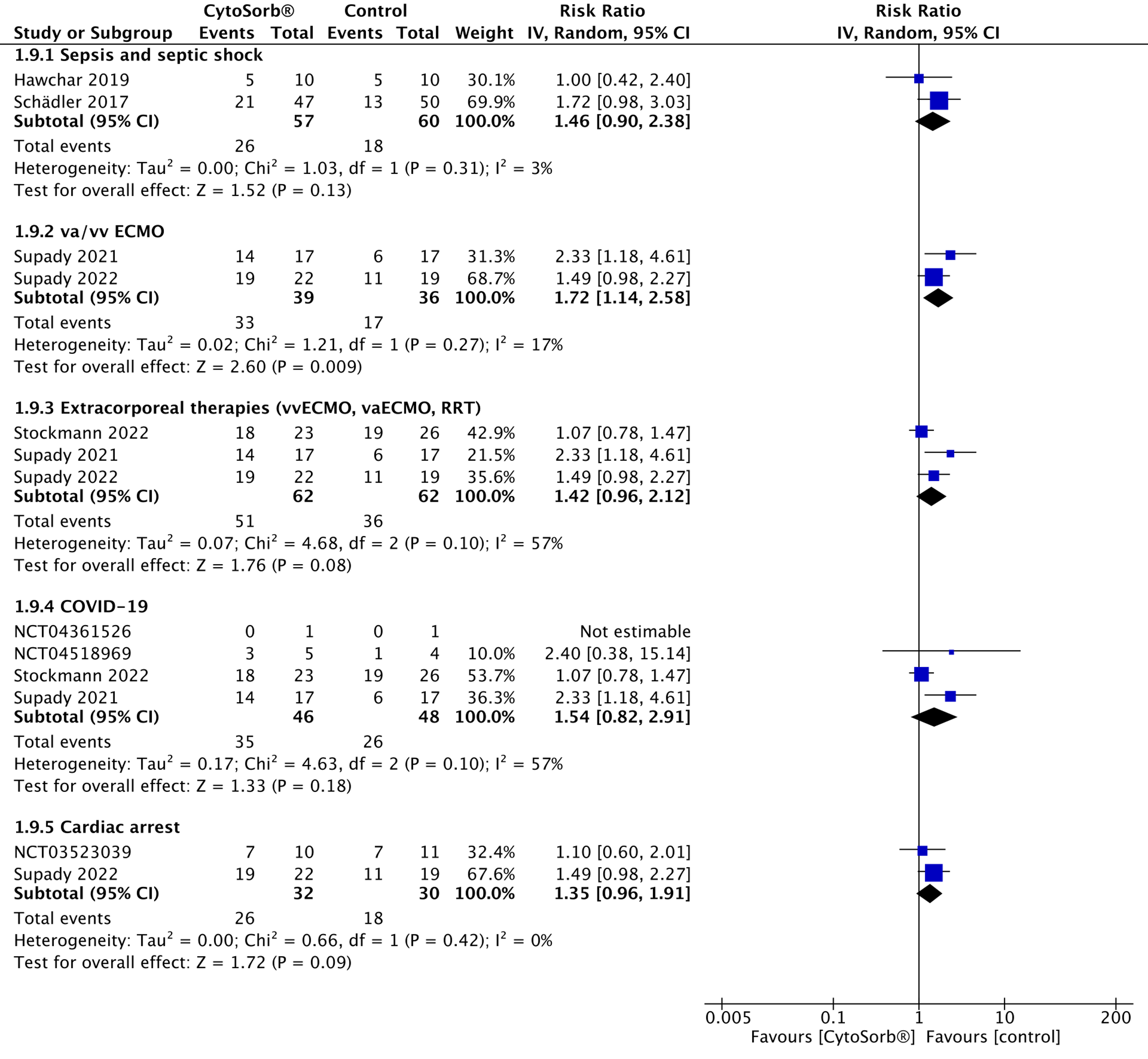
**

# **Supplementary Figure S4 – Primary outcome: funnel plot.**

#

# **
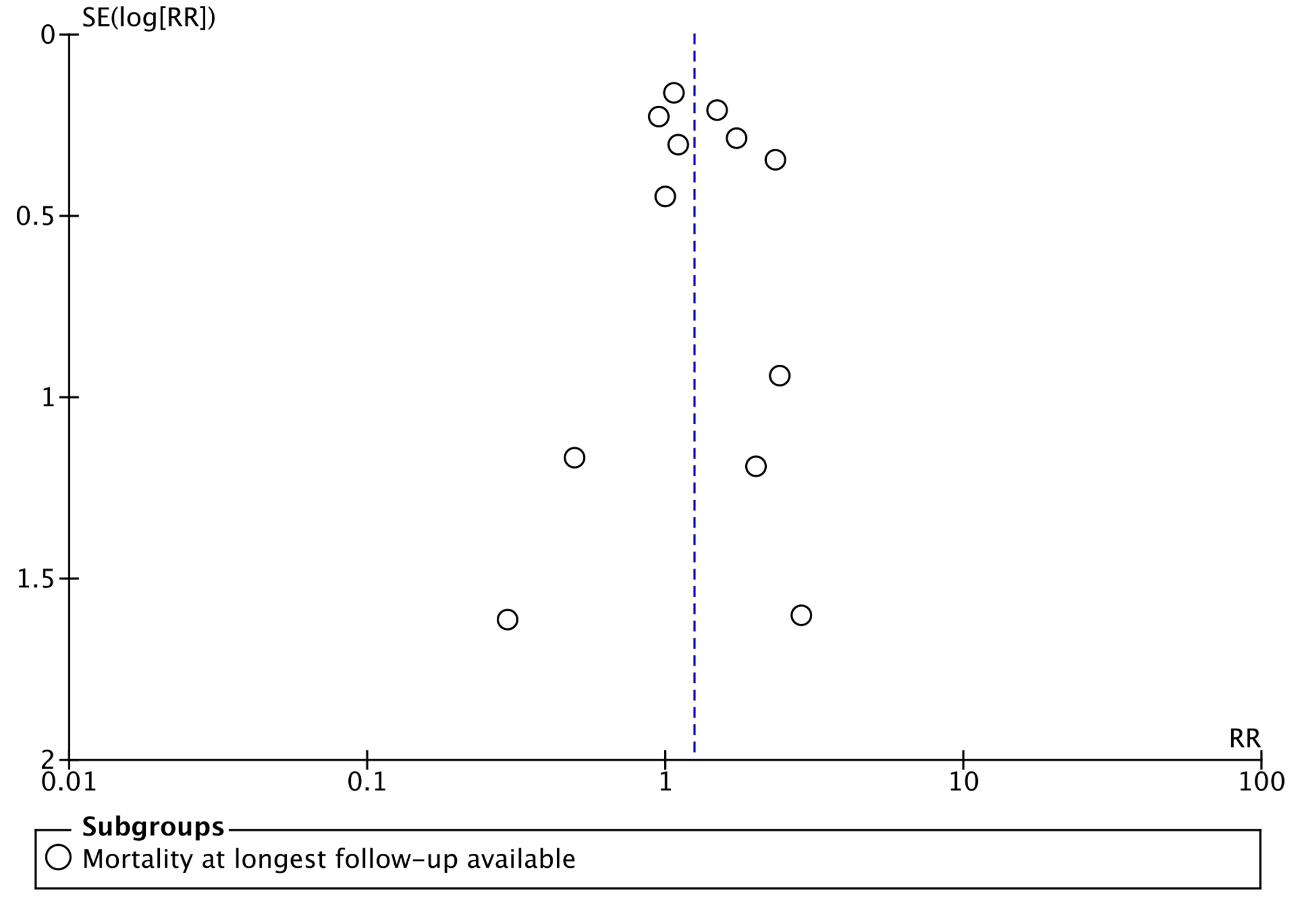
**

# **Supplementary Table S6 – Certainty of the body of evidence assessment using the grading of recommendations assessment, development and evaluation (GRADE) framework: primary and secondary outcomes.**

| **Certainty assessment** | | | | | | | **№ of patients** | | **Effect** | | **Certainty** | **Importance** |
| --- | --- | --- | --- | --- | --- | --- | --- | --- | --- | --- | --- | --- |
| **№ of studies** | **Study design** | **Risk of bias** | **Inconsistency** | **Indirectness** | **Imprecision** | **Other considerations** | **CytoSorb** | **control** | **Relative (95% CI)** | **Absolute (95% CI)** |  |  |
| **Mortality at longest follow-up available** | | | | | | | | | | | | |
| 16 | randomised trials | serious^a^ | not serious | not serious | serious^b^ | none | 120/402 (29.9%) | 98/405 (24.2%) | **RR 1.24** (1.04 to 1.49) | **6 more per 100** (from 1 more to 12 more) | ⨁⨁◯◯ Low | CRITICAL |
| **Mortality at 30-days** | | | | | | | | | | | | |
| 11 | randomised trials | serious^a^ | not serious | not serious | serious^b^ | none | 100/361 (27.7%) | 75/366 (20.5%) | **RR 1.41** (1.06 to 1.88) | **8 more per 100** (from 1 more to 18 more) | ⨁⨁◯◯ Low | CRITICAL |
| **Patients with at least one serious adverse event** | | | | | | | | | | | | |
| 4 | randomised trials | very serious^c^ | serious^d^ | not serious | very serious^e^ | none | 50/104 (48.1%) | 33/106 (31.1%) | **RR 1.42** (0.87 to 2.33) | **13 more per 100** (from 4 fewer to 41 more) | ⨁◯◯◯ Very low | IMPORTANT |
| **Patients with at least one adverse event** | | | | | | | | | | | | |
| 6 | randomised trials | very serious^c^ | serious^d^ | not serious | very serious^f^ | none | 85/147 (57.8%) | 79/152 (52.0%) | **RR 1.09** (0.98 to 1.21) | **5 more per 100** (from 1 fewer to 11 more) | ⨁◯◯◯ Very low | IMPORTANT |
| **Adverse event leading to death** | | | | | | | | | | | | |
| 2 | randomised trials | very serious^c^ | serious^d^ | not serious | very serious^e^ | none | 3/38 (7.9%) | 3/38 (7.9%) | **RR 0.99** (0.19 to 5.06) | **0 fewer per 100** (from 6 fewer to 32 more) | ⨁◯◯◯ Very low | CRITICAL |
| **Total number of serious adverse events** | | | | | | | | | | | | |
| 3 | randomised trials | very serious^c^ | not serious | not serious | very serious^g^ | none | 83/85 | 70/88 | **Rate ratio 1.18** (0.86 to 1.63) | **-** | ⨁◯◯◯ Very low | IMPORTANT |
| **Total number of adverse events** | | | | | | | | | | | | |
| 6 | randomised trials | very serious^c^ | serious^d^ | not serious | very serious^g^ | none | 623/256 | 628/268 | **Rate ratio 0.99** (0.86 to 1.15) | **-** | ⨁◯◯◯ Very low | IMPORTANT |
| **Total number of non-serious adverse events** | | | | | | | | | | | | |
| 3 | randomised trials | very serious^c^ | not serious | not serious | very serious^g^ | none | 114/85 | 145/88 | **Rate ratio 0.79** (0.62 to 1.01) | **-** | ⨁◯◯◯ Very low | IMPORTANT |
| **Total number of device-related adverse events** | | | | | | | | | | | | |
| 6 | randomised trials | very serious^c^ | not serious | not serious | very serious^g^ | none | 10/124 | 0/122 | **Rate ratio 2.90** (0.70 to 12.05) | **-** | ⨁◯◯◯ Very low | IMPORTANT |

**CI:** confidence interval; **RR:** risk ratio

#### Explanations

a. Some trials with high-risk of bias and lacking an intention-to-treat analysis.

b. TSA-adjusted confidence interval crossing a relative risk of 1.00.

c. No trial with low risk of bias in adverse events outcomes. Several trials lacking an intention-to-treat analysis. Most of the trials lacking outcome definition. Some trials excluding patients presenting adverse events.

d. Significant statistical heterogeneity (I-squared > 50%, p-value for heterogeneity ≤ 0.10)

e. Underreporting of the outcome and very little information size at trial sequential analysis.

f. Underreporting of the outcome and TSA-adjusted confidence interval crossing a relative risk of 1.00.

g. Underreporting of the outcome. Low cumulative information size and wide 95% confidence interval.

# **Supplementary Results S1 – Adverse events reporting.**

Ten trials (n=640) reported adverse event data (Schadler 2017, Poli 2019, Gleason 2019, Bernardi 2016, Holmén 2022, Hawchar 2019, Asch 2021, Diab 2022, Garau 2019, Stockmann 2022). Only 1 trial reported the definition of SAE (Poli 2019); no trial defined AE, device-related AE, or AE leading to death. No trial graded AEs according to the Common Terminology Criteria for Adverse Events (Available from: <https://ctep.cancer.gov/protocoldevelopment/electronic_applications/ctc.htm>). Results on AEs, SAEs and device-related AEs were reported in 4 trials (Schadler 2017, Poli 2019, Gleason 2019, Bernardi 2016), while 2 trials reported only number of AEs (Asch 2021, Diab 2022), 1 trial AEs and AE leading to death (Garau 2019), 1 trial only AEs (Stockmann 2022), and 2 trials only device-related AEs (Holmén 2022, Hawchar 2019). Two trials found zero AEs in both groups (Garau 2019, Bernardi 2016).

One trial (n=20) excluded patients with recurrent circuit clotting (n=2) and hypotension (n=2) related to the extracorporeal therapy (CytoSorb® combined with hemodialysis) from the analysis (Asch 2021). Two trials excluded patients with shorter or longer CPB duration from the primary analysis (Garau 2019, Taleska Stupica).

# **Supplementary Figure S5 – Secondary outcomes: number of adverse events**

**
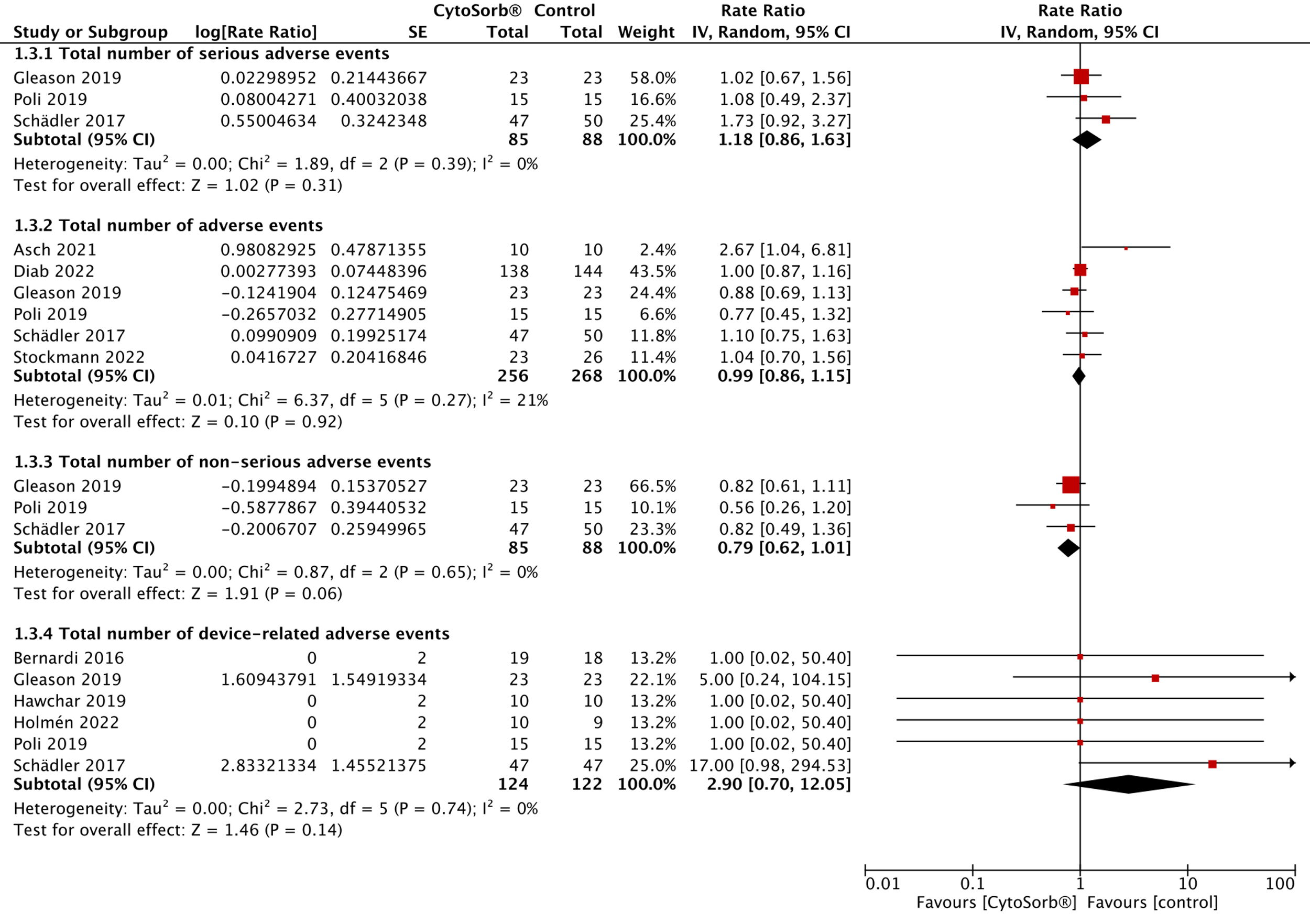
**
